# The Effects of Combined Caffeine and Sodium Bicarbonate Supplementation on Exercise Performance, Physiological Responses, and Gastrointestinal Tolerability: A Systematic Review and Meta-Analysis

**DOI:** 10.3390/nu18193171

**Published:** 2026-09-26

**Authors:** Xiang Chen, Wenhui Tu, Jun Wang

**Affiliations:** 1 School of Sport Science, Beijing Sport University, Beijing 100084, China; 2025210352@bsu.edu.cn (X.C.); 2025210350@bsu.edu.cn (W.T.); 2 Laboratory of Sports Stress and Adaptation, General Administration of Sport, Beijing 100084, China

**Keywords:** caffeine, sodium bicarbonate, combined supplementation, exercise performance, physiological responses, gastrointestinal tolerability

## Abstract

**Background:** Caffeine and sodium bicarbonate can individually enhance exercise performance, but whether their combined use provides additional benefits and how it affects physiological responses and gastrointestinal (GI) tolerability remain unclear. **Objective:** This systematic review and meta-analysis aimed to evaluate the effects of combined caffeine and sodium bicarbonate (CAF + SB) supplementation on exercise performance, physiological responses, and GI tolerability, and to determine whether it provides additional benefits compared with placebo or either supplement alone. **Methods:** PubMed, Web of Science, and SPORTDiscus were systematically searched from inception to 18 July 2026. Continuous outcomes were pooled using random-effects models and expressed as mean differences (MDs) or ratios of means (RoMs) with 95% confidence intervals (CIs), as appropriate. Within-participant correlations in crossover trials were accounted for, and robustness was examined using leave-one-out analyses and alternative correlation coefficients. Rating of perceived exertion (RPE) and GI symptoms were synthesized narratively. **Results:** Thirteen crossover trials were included. Compared with placebo, CAF + SB supplementation increased mean power (MD = 8.43 W, 95% CI = 1.85 to 15.00, *p* = 0.012) and reduced time-trial completion time (RoM = 0.984, 95% CI = 0.970 to 0.998, *p* = 0.022), corresponding to an estimated reduction of 1.6%. However, both findings were sensitive to the exclusion of individual studies in leave-one-out analyses, indicating limited robustness. CAF + SB did not significantly improve mean power compared with caffeine or sodium bicarbonate alone. Compared with placebo, CAF + SB increased blood HCO_3_^−^ and post-exercise lactate concentrations, although the HCO_3_^−^ evidence was limited by heterogeneity or few studies. Narrative findings showed no consistent effect on RPE or GI tolerability. **Conclusions:** CAF + SB supplementation may produce modest improvements in selected exercise-performance outcomes compared with placebo, but these findings are not robust because their statistical significance depends on individual studies. Current evidence does not establish an additional performance benefit over caffeine or sodium bicarbonate alone. The observed physiological responses do not, by themselves, demonstrate superior performance, and the gastrointestinal tolerability of CAF + SB remains uncertain.

## 1. Introduction

Nutritional strategies for optimizing exercise performance have long focused on ensuring adequate energy availability, optimizing the metabolic environment, and promoting recovery and adaptation, while sports supplements are commonly used to further enhance performance in specific exercise contexts [1,2]. Caffeine and sodium bicarbonate are among the most extensively investigated ergogenic supplements [3,4], but they act through distinct physiological pathways. Caffeine primarily antagonizes adenosine A_1_ and A_2_A receptors in the central nervous system, potentially influencing perceived exertion, pain, central drive, and neuromuscular function [3,5,6]. Sodium bicarbonate, in contrast, increases extracellular bicarbonate availability and buffering capacity, thereby increasing the transmembrane gradient that favors H^+^ efflux from active muscle and potentially supporting performance during high-intensity exercise with substantial glycolytic demands [7,8,9].

These distinct mechanisms provide a physiological rationale for CAF + SB supplementation. Any additional benefit would be biologically plausible during high-intensity exercise in which substantial glycolytic and acid–base disturbances coexist with central, perceptual, or neuromuscular constraints. Nevertheless, mechanistic complementarity does not necessarily produce additive improvements in performance, because fatigue is determined by multiple interacting processes that vary according to exercise modality, intensity, and duration [9,10,11]. Exercise characteristics should therefore be considered a potential source of variation in the effects of CAF + SB rather than an established effect modifier.

Participant characteristics and supplementation protocols may further influence responses to CAF + SB supplementation [12,13]. Training status may affect exercise capacity, metabolic responses, and responsiveness to supplementation [3,4], whereas dose, formulation and intake timing may influence both physiological responses and GI tolerability [14,15,16]. GI discomfort may also offset a potential performance benefit, particularly when SB is administered in a large acute dose (0.4 or 0.5 g/kg) [4,17]. Accordingly, performance outcomes should be interpreted alongside physiological responses, RPE, and GI symptoms.

Individual studies of CAF + SB have produced inconsistent findings [18,19,20,21]. Grgic [12] critically reviewed eight trials of isolated and combined CAF and SB supplementation, but did not quantitatively pool their effects and found limited evidence of an additive performance benefit. More recently, Zart and Fröhlich [22] evaluated several supplement combinations in endurance exercise lasting longer than 35 s; their CAF + SB analyses did not demonstrate statistically significant benefits over placebo, CAF alone, or SB alone. Luo et al. [23] used a Bayesian network meta-analysis to compare numerous dietary supplements in elite combat-sport athletes, thereby addressing a population- and sport-specific question rather than the effects of CAF + SB across broader exercise contexts. Consequently, a dedicated quantitative synthesis that distinguishes the overall effect of CAF + SB relative to placebo from its potential additional benefit over each constituent alone, while also integrating physiological and tolerability outcomes, remains warranted.

Accordingly, this systematic review and meta-analysis aimed to evaluate the effects of CAF + SB supplementation on exercise performance, physiological responses, and GI symptoms relative to placebo, and to determine whether it provides additional performance benefits beyond those of caffeine or sodium bicarbonate alone.

## 2. Methods

This systematic review and meta-analysis followed the PRISMA 2020 statement [24]. The study protocol was registered with PROSPERO (CRD420261452988).

### 2.1. Search Strategy

A systematic literature search was conducted in PubMed, Web of Science, and SPORTDiscus from database inception through 18 July 2026. The search strategy was developed around two core concepts: caffeine and sodium bicarbonate. Search terms were adapted to the indexing systems and search field characteristics of each database. Within each concept, related terms were combined using the Boolean operator “OR” whereas the two concepts were combined using “AND” (Supplementary File S1). The electronic search was limited to these three bibliographic databases, and trial registries were not searched. To supplement the database search, backward and forward citation searching was performed by manually screening the reference lists of the included studies and conducting citation searches for each included study.

### 2.2. Study Selection and Eligibility Criteria

After the literature search was completed, all retrieved records were imported into EndNote 2025.3 for reference management and deduplication. Following automated deduplication, two reviewers (X.C. and W.T.) manually checked the remaining records and independently screened titles, abstracts, and subsequently eligible full texts. Disagreements were resolved through discussion, with a third reviewer consulted when necessary.

Eligibility criteria were defined according to the PICOS (Participants, Intervention, Comparator, Outcomes, and Study Design) framework: (1) Participants: Healthy adults aged ≥18 years who regularly engaged in physical activity or competitive sports, regardless of sex; (2) Intervention: Studies evaluating the CAF + SB supplement were eligible, with no restrictions on the dose, administration timing, or formulation of either supplement; (3) Comparator: Studies were required to include at least one placebo or control condition, or a caffeine- or sodium bicarbonate-only condition; (4) Outcomes: At least one of the following outcome categories was required: exercise performance measures, including mean power, time-trial completion time, and other performance-related outcomes; physiological responses, including blood lactate concentration, blood HCO_3_^−^ concentration, and blood pH; subjective perceptions, including RPE; tolerability measures, including GI symptoms; (5) Study design: Randomized controlled crossover trials. Studies without an eligible CAF + SB intervention or comparator condition were excluded. Studies involving additional supplements were excluded when the effect of CAF + SB could not be isolated. Non-randomized studies, reviews, conference abstracts, theses, non-English publications, and studies for which the full text was unavailable or relevant outcome data could not be extracted were excluded. The English-language restriction was prespecified in the registered protocol and reflected the unavailability of resources for the reliable translation and duplicate assessment of non-English reports.

### 2.3. Data Extraction and Transformation

After study screening was completed, two reviewers independently extracted data using a standardized Microsoft Excel worksheet (Microsoft Excel, version 16.111.2). The extracted data included: (1) study characteristics (first author, publication year, and country); (2) participant characteristics (sample size, age, sex, body weight, and training background); (3) exercise protocols (sport and test protocol); (4) supplementation protocols (caffeine dose, sodium bicarbonate dose, timing of intake); (5) outcome measures (exercise performance, physiological responses, RPE, and GI symptoms).

For quantitative synthesis, we extracted the sample size for each comparison, condition-specific means and standard deviations (SDs), and any directly reported paired or model-adjusted effect estimates with their corresponding SDs, standard errors (SEs), or CIs. Variance estimates were obtained hierarchically from: (1) a reported SD or SE of the paired effect; (2) an SE calculated from the CI of a paired or model-adjusted effect; or (3) condition-specific SDs using an assumed within-participant correlation of r = 0.50 when paired variance was unavailable. The variance source used for each study–outcome–comparison is detailed in Supplementary File S2.

When data were available only in graphical form, WebPlotDigitizer 4.7 was used for data extraction [25]. For studies reporting the MDs and 95% CIs without a corresponding SE, the SE was calculated from the reported 95% CI using Equation (1) [26]:

(1) $$SE=\frac{\left(Upper-Lower\right)}{2\times 1.96}$$

When a study reported outcome data separately for men and women, the sex-specific data were pooled when the intervention, outcome measure, and measurement time point were consistent, following the methods recommended in the Cochrane Handbook to avoid double-counting participants from the same study in the meta-analysis (Equation (2)) [26]:

(2) $${SD}_{combined}=\sqrt{\frac{(N_{1}-1){{SD}_{1}}^{2}+(N_{2}-1){{SD}_{2}}^{2}+\frac{N_{1}N_{2}}{N_{1}+N_{2}}\times {\left({Mean}_{1}-{Mean}_{2}\right)}^{2}}{N_{1}+N_{2}-1}}$$

### 2.4. Assessment of Risk of Bias

Two reviewers independently assessed the risk of bias of each included study using the Cochrane Risk of Bias 2 (RoB 2) tool for randomized crossover trials [27]. The assessment covered six domains: bias arising from the randomization process (D1), bias arising from period and carryover effects (Domain S), bias due to deviations from intended interventions (D2), bias due to missing outcome data (D3), bias in measurement of the outcome (D4), and bias in selection of the reported result (D5). Domain-level and overall risk-of-bias judgments were classified as “low risk of bias,” “some concerns,” or “high risk of bias” according to the relevant signalling questions and RoB 2 decision algorithms. Before consensus, inter-rater agreement was evaluated using percentage agreement and unweighted Cohen’s kappa (κ) based on the two reviewers’ independent assessments. Any disagreements were resolved through discussion, with a third researcher consulted when consensus could not be reached.

### 2.5. Statistical Analysis

Meta-analyses were performed in R (version 4.6.0) using the metafor package (version 5.0.1) [28]. Outcomes were pooled when sufficiently comparable in construct, measurement scale, and assessment timing. Random-effects models were fitted using inverse-variance weighting, restricted maximum likelihood (REML) estimation, and Wald-type 95% CIs.

MDs were used for outcomes reported in comparable units, including absolute mean power (W), blood lactate concentration (mmol/L), and blood bicarbonate concentration (mmol/L). Absolute mean-power outcomes reported in watts were pooled as an exploratory cross-protocol synthesis; however, the common measurement unit was not considered to imply physiological equivalence across exercise tasks.

Because the time-trial protocols differed substantially in distance and completion time, time-trial performance was analyzed using ratios of means (RoMs) rather than MDs. This change was introduced during peer review and was not prespecified in the registered protocol. RoMs were pooled on the logarithmic scale and back-transformed for presentation. The corresponding percentage reduction in completion time was calculated using Equation (3) [29]:

(3) (1−RoM)×100%

For crossover trials, directly reported paired effect estimates and corresponding SEs or CIs were used preferentially. When paired variance information was unavailable, the standard deviation of the paired differences was calculated using Equation (4), as recommended in the Cochrane Handbook [26]:

(4) $${SD}_{diff}=\sqrt{{{SD}_{1}}^{2}+{{SD}_{2}}^{2}-2r{SD}_{1}{SD}_{2}}$$

The corresponding SE was calculated using Equation (5):

(5) $$SE=\left(SD_{diff}/\sqrt{n}\right)$$

An assumed within-participant correlation of *r* = 0.50 was used in the primary analysis, with *r* = 0.30 and 0.70 examined in sensitivity analyses [26]. Correlation imputation was applied only when no directly usable paired or model-adjusted variance estimate was available.

Statistical heterogeneity was evaluated using Cochran’s Q, τ^2^, and I^2^ [30,31]. Robustness was examined, where applicable, using alternative within-participant correlations and leave-one-out analyses. For the placebo-controlled analyses of mean power and time-trial completion time, study weights, standard influence diagnostics, and exploratory Riley-type 95% prediction intervals were additionally examined. RPE and GI symptoms were synthesized narratively because of substantial variation in measurement instruments, assessment time points, and reporting formats. Funnel plots and Egger’s regression tests were not performed because all meta-analyses included fewer than 10 studies [32]. All statistical tests were two-sided, with *p* < 0.05 indicating statistical significance.

### 2.6. Assessment of Evidence Certainty

The Grading of Recommendations Assessment, Development and Evaluation (GRADE) approach was used to assess the certainty of evidence across five domains [33]: risk of bias, inconsistency, indirectness, imprecision, and publication bias. Two researchers independently rated the certainty of evidence as high, moderate, low, or very low. Disagreements were resolved through discussion, with a third researcher consulted when consensus could not be reached.

## 3. Results

### 3.1. Search Results

A total of 412 records were identified, including 182 from PubMed, 155 from Web of Science, and 75 from SPORTDiscus. After deduplication, 273 records underwent title and abstract screening, and 21 full-text reports were assessed for eligibility. Eight full-text reports were excluded, primarily because of duplicate publications, conference abstracts, or the absence of a CAF + SB supplementation intervention. Additionally, three potentially relevant reports were identified through reference and citation searching; however, none met the inclusion criteria. Ultimately, 13 randomized controlled crossover trials were included in the review. The study selection process is presented in Figure 1.

### 3.2. Basic Characteristics of the Included Studies

The 13 included studies were randomized, double-blind, placebo-controlled crossover trials published between 2008 and 2024. The sample size ranged from 6 to 28 participants per study, with a total of 192 participants (152 men and 40 women) aged 20.5–35.2 years; two studies did not report participant age. Participants ranged from recreationally active individuals to trained and elite athletes [18,19,20,21,34,35,36,37,38,39,40,41,42]. The exercise protocols were heterogeneous and included single or repeated sprint tests, cycling, rowing, and swimming time trials, time-to-exhaustion tests, and resistance exercise protocols. The sodium bicarbonate dose was 0.3 g/kg in all included studies and was administered 60–120 min before exercise. Seven studies used a split-dose strategy, and sodium bicarbonate was administered in capsule or solution form. Caffeine doses ranged from 3.0 to 6.2 mg/kg and were administered 30–60 min before exercise. Reported washout intervals ranged from 48 h to 7 days, while one study did not clearly report the interval. Detailed participant characteristics, exercise protocols, supplementation strategies and washout intervals are presented in Table 1.

### 3.3. Risk of Bias in the Included Studies

Regarding the randomization process (D1), 10 studies (76.9%) were judged to have some concerns, mainly because of insufficient reporting of the randomization procedures. Eight studies (61.5%) had some concerns regarding bias arising from period and carryover effects (Domain S). All 13 studies were judged to have a low risk of bias due to deviations from intended interventions (D2) and missing outcome data (D3). Regarding outcome measurement (D4), 12 studies were judged to have a low risk of bias, while Rezaei et al. [34] had some concerns. Regarding selection of the reported result (D5), 11 studies (84.6%) raised some concerns, primarily because trial registration or prespecified analysis plans were unavailable. Overall, all 13 studies were judged to have some concerns, and none was judged to have a high risk of bias. Before consensus, raw agreement between the two reviewers ranged from 23.1% to 100.0%, while Cohen’s kappa values for the estimable domains ranged from −0.121 to 0.350. Detailed study-level judgments, consensus rationales, and inter-rater agreement results are provided in Supplementary File S3.

### 3.4. Meta-Analysis

#### 3.4.1. Mean Power

Six studies were included to evaluate the effect of CAF + SB supplementation on mean power compared with placebo. One additional study reported only relative mean power (W·kg^−1^) [18], which could not be pooled with absolute mean power (W) and was therefore excluded from this analysis.

Compared with placebo, CAF + SB supplementation significantly increased mean power (MD = 8.43 W, 95% CI = 1.85 to 15.00, *p* = 0.012, I^2^ = 0%). However, this finding was sensitive to the inclusion of Moesgaard et al. [39], as the pooled effect was no longer statistically significant when this study was excluded in the leave-one-out analysis. Compared with caffeine supplementation alone (4 studies), CAF + SB supplementation had no significant effect on mean power (MD = −2.91 W, 95% CI = −20.00 to 14.18, *p* = 0.738, I^2^ = 0%). Similarly, compared with sodium bicarbonate supplementation alone (5 studies), CAF + SB supplementation did not significantly increase mean power (MD = 6.49 W, 95% CI = −7.98 to 20.96, *p* = 0.379, I^2^ = 0%). The primary analyses are presented in Figure 2, and detailed leave-one-out and correlation sensitivity analyses are reported in Section 3.5 and Supplementary Files S4 and S5.

#### 3.4.2. Time-Trial Completion Time

Four studies evaluating the effect of CAF + SB supplementation on time-trial completion time were included. Studies using a progressive workload-to-exhaustion test (Rezaei et al. [34] and Higgins et al. [42]) were not included in the quantitative meta-analysis of this outcome because time to exhaustion differs from time-trial completion time. Compared with placebo, CAF + SB supplementation significantly reduced time-trial completion time (RoM = 0.984, 95% CI = 0.970 to 0.998, *p* = 0.022, I^2^ = 0%), corresponding to an estimated 1.6% shorter completion time (95% CI: 0.2–3.0% shorter). However, this finding was sensitive to the exclusion of either Correia-Oliveira and Lopes-Silva or Pruscino et al. [20,37]. The pooled effect was no longer statistically significant when either study was omitted in the leave-one-out analyses. The primary analysis is presented in Figure 3, and detailed leave-one-out and correlation sensitivity analyses are reported in Section 3.5 and Supplementary Files S4 and S5.

#### 3.4.3. Physiological Responses

##### Blood Lactate

Four studies evaluated the effects of CAF + SB supplementation on post-exercise blood lactate concentrations, measured at exercise cessation or immediately after exercise in three studies and at 2 min post-exercise in Carr et al. (Figure 4). Compared with placebo, CAF + SB supplementation was associated with significantly higher blood lactate concentrations (MD = 4.18 mmol/L, 95% CI = 3.34 to 5.03; *p* < 0.001; I^2^ = 0%).

##### Blood HCO_3_^−^

Four studies assessed pre-exercise blood HCO_3_^−^ concentrations (Figure 5). Compared with placebo, CAF + SB supplementation was associated with significantly higher pre-exercise blood HCO_3_^−^ concentrations (MD = 6.16 mmol/L, 95% CI = 4.85 to 7.48; *p* < 0.001), with substantial heterogeneity (I^2^ = 79.3%). Sensitivity analyses showed that the pooled effect remained statistically significant and the direction of the effect was unchanged when any single study was excluded, although substantial heterogeneity persisted (I^2^ = 71.9–86.4%). Two studies assessed post-exercise blood HCO_3_^−^ concentrations, showing a significantly higher concentration with CAF + SB supplementation than with placebo (MD = 1.82 mmol/L, 95% CI = 1.16 to 2.48; *p* < 0.001; I^2^ = 0%). Given the small number of studies, these pooled results should be interpreted with caution. The results are presented in Figure 5.

#### 3.4.4. RPE

Of the 13 included studies, 10 assessed RPE. Differences in measurement scales, assessment time points, and exercise protocols precluded quantitative pooling; therefore, the findings were synthesized narratively. Seven studies reported inferential between-condition analyses: Rezaei et al. [34] and Ziyaiyan et al. [41] reported lower RPE with CAF + SB in selected comparisons or at specific time points, whereas five studies found no statistically significant treatment effects. Two studies provided condition-specific values without corresponding between-condition statistical comparisons, and one did not report RPE outcome data. Overall, the available evidence did not demonstrate a consistent effect of CAF + SB on RPE. Detailed findings are presented in Table 2.

#### 3.4.5. Gastrointestinal Symptoms

Twelve of the 13 included studies assessed GI symptoms. Differences in assessment methods, symptoms evaluated, measurement time points, and reporting formats precluded quantitative pooling; therefore, the findings were synthesized narratively according to the reported comparator.

For CAF + SB versus placebo, four studies reported greater GI symptoms or discomfort with CAF+SB: Montalvo-Alonso et al. [21] (*p* = 0.029), Ferragut et al. [18] (*p* = 0.021), Ziyaiyan et al. [41] (exact *p* value unavailable), and Higgins et al. [42] (*p* < 0.01). Five studies reported no statistically significant overall treatment or between-condition effect; however, comparator-specific pairwise results were not consistently available. For CAF + SB versus caffeine alone, Montalvo-Alonso et al. [21] and Ferragut et al. [18] reported greater GI discomfort with CAF + SB (*p* = 0.011 and *p* = 0.005, respectively). No study provided an interpretable pairwise statistical comparison of CAF+SB with sodium bicarbonate alone or with a control condition.

Findings involving sodium bicarbonate alone were considered separately. Montalvo-Alonso et al. [21] and Ferragut et al. [18] reported greater GI discomfort with sodium bicarbonate than with caffeine or placebo, while Ziyaiyan et al. [41] and Higgins et al. [42] reported greater symptoms with sodium bicarbonate than with placebo. Pruscino et al., Carr et al., and Moesgaard et al. [37,38,39] provided descriptive findings without formal between-condition comparisons and were therefore not interpreted as evidence of comparator-specific differences. Overall, the available evidence did not support a consistent conclusion regarding the comparative GI tolerability of CAF + SB. Detailed findings are presented in Table 3.

### 3.5. Sensitivity and Influence Analyses

Leave-one-out analyses indicated that the significant effect of CAF + SB versus placebo on mean power was sensitive to the inclusion of Moesgaard et al. [39]; after this study was excluded, the pooled effect was no longer statistically significant (MD = 9.85 W, 95% CI = −3.82 to 23.53, *p* = 0.160, I^2^ = 0%). The time-trial result was also sensitive to study omission. The pooled effect became non-significant after exclusion of Correia-Oliveira and Lopes-Silva (RoM = 0.985, 95% CI = 0.970 to 1.001, *p* = 0.067, I^2^ = 0%) or Pruscino et al. (RoM = 0.987, 95% CI = 0.969 to 1.005, *p* = 0.148, I^2^ = 0%) [20,37]. Other applicable leave-one-out analyses did not alter the direction or statistical conclusions of the pooled estimates (Supplementary File S4).

Influence diagnostics flagged Moesgaard et al. in the mean-power model, reflecting its dominant model weight (76.87%) and high leverage (hat value = 0.769), rather than an extreme studentized residual. No time-trial study was formally flagged, although Pruscino et al. received the largest model weight (41.31%). The exploratory 95% prediction intervals crossed the null value for both mean power (−0.89 to 17.74 W) and time-trial completion time (RoM = 0.954 to 1.015) (Supplementary File S4).

Sensitivity analyses using alternative within-participant correlations of r = 0.30 and r = 0.70 produced similar effect directions and magnitudes across outcomes. Statistical conclusions remained unchanged except for time-trial performance, which was no longer statistically significant at r = 0.30 (*p* = 0.052) but remained significant at r = 0.70 (*p* = 0.004). Complete sensitivity-analysis results are presented in Supplementary File S5.

### 3.6. GRADE Evidence Assessment

The certainty of evidence ranged from very low to moderate across outcomes and comparisons (Supplementary File S6). The certainty assessments considered the total information size and confidence-interval width, crossover-specific period and carryover concerns, incomplete reporting, and the robustness of the pooled estimates to study omission and alternative correlation assumptions. Moderate-certainty evidence was identified for blood lactate concentration. The certainty of evidence was low for mean power across all three comparisons, time-trial performance, and pre- and post-exercise blood bicarbonate concentrations, primarily because of concerns regarding risk of bias, imprecision, and, for pre-exercise bicarbonate, substantial unexplained inconsistency. The certainty of evidence for RPE and GI symptoms was very low because of risk-of-bias concerns, inconsistent findings, heterogeneous assessment methods, and incomplete or imprecise reporting.

## 4. Discussion

The principal finding of this systematic review and meta-analysis was that CAF + SB supplementation was associated with modest improvements in mean power and time-trial completion time compared with placebo. However, these performance findings should be interpreted cautiously because their statistical significance was sensitive to the exclusion of individual studies or, for time-trial performance, the assumed within-participant correlation. CAF + SB supplementation was also associated with higher blood bicarbonate and post-exercise blood lactate concentrations, whereas no consistent effect on RPE was observed and GI findings varied across studies, with greater discomfort reported under CAF + SB in some studies. Moreover, CAF + SB did not provide a clear additional performance benefit over caffeine or sodium bicarbonate alone; therefore, the available evidence does not establish an additional effect of co-supplementation.

From a mechanistic perspective, caffeine and sodium bicarbonate may influence exercise performance through distinct pathways. Caffeine primarily acts through central adenosine-receptor antagonism and may influence perceived exertion, central drive, and neuromuscular function [43,44,45]. In contrast, sodium bicarbonate increases extracellular buffering capacity by elevating blood bicarbonate concentrations, which may facilitate acid–base regulation and attenuate fatigue during high-intensity exercise [7,46]. These mechanisms provide a rationale for investigating co-supplementation, but the present meta-analysis did not directly test whether they mediated the observed performance differences. Nevertheless, CAF + SB did not show a clear advantage for mean power over caffeine or sodium bicarbonate alone, consistent with the lack of an additional benefit reported by Zart and Fröhlich [22]. Moreover, the pairwise comparisons included in the present meta-analysis were not formal tests of statistical interaction. Accordingly, physiological complementarity should not be interpreted as evidence of an additive performance effect. For the same reason, the available direct comparisons were insufficient to determine whether a synergistic interaction was present or absent.

The effects of CAF + SB may also depend on the physiological demands and principal limiting factors of the exercise task. Sodium bicarbonate may be particularly relevant during high-intensity exercise in which glycolytic flux and H^+^ accumulation contribute substantially to fatigue [8,47,48], whereas caffeine may be more influential when central drive or neuromuscular function limits performance [43,44]. Targeting both pathways could therefore be advantageous under selected exercise conditions, but this remains a hypothesis. The small number of available studies precluded reliable subgroup or meta-regression analyses of exercise type, duration, or training status.

To further interpret the observed performance findings, this study examined blood acid–base and metabolic responses. Compared with placebo, CAF + SB supplementation increased pre-exercise blood HCO_3_^−^ concentrations, although substantial between-study heterogeneity was observed (I^2^ = 79.3%). This group-level increase does not establish individual peak alkalosis, which may vary with dosing and sampling schedules, formulation, meal conditions, baseline status, and time to peak. Post-exercise blood HCO_3_^−^ concentrations were also higher following CAF + SB; however, this analysis included only two studies and should therefore be interpreted cautiously. These findings are consistent with the expected effect of sodium bicarbonate on extracellular acid–base status, whereby increased bicarbonate availability may strengthen the transmembrane H^+^ gradient and facilitate H^+^ efflux from working muscle [4,9]. CAF + SB supplementation also increased early post-exercise blood lactate concentrations, consistent with the findings of a previous meta-analysis by Luo et al. [23]. However, higher post-exercise blood lactate should not be taken as evidence of greater glycolytic flux or fatigue, because its concentration reflects production, transport, uptake, and clearance and may also vary with work performed, exercise intensity, and sampling time. Increased extracellular buffering could facilitate the transport of H^+^ and lactate from working muscle and may permit greater glycolytic energy provision during high-intensity exercise [8,49]. Nevertheless, this explanation remains speculative because blood HCO_3_^−^ and lactate concentrations cannot directly establish changes in intramuscular pH, glycolytic flux, or the mechanisms responsible for the observed performance effects. Because the blood responses were primarily compared with placebo, they cannot establish a greater physiological effect of CAF + SB than of either supplement alone. Even if such an effect occurred, its performance relevance might depend on whether acid–base regulation limits the exercise task; this possibility was not tested. Therefore, future studies incorporating direct measurements of intramuscular acid–base status and metabolic flux are required to determine whether these physiological responses contribute meaningfully to performance.

Findings for RPE were inconsistent and did not indicate that CAF + SB supplementation reliably reduced perceived exertion. Although caffeine may influence perceptions of effort, fatigue, and pain through its central actions, RPE is a multidimensional response affected by exercise mode and intensity, assessment timing, scale characteristics, and individual experience [50,51,52]. Among the seven studies that provided inferential comparisons, lower RPE was reported in only two studies and was limited to selected comparisons or assessment points, whereas five studies found no statistically significant treatment effect. Consequently, the available evidence cannot determine whether these inconsistent findings reflect genuine task-dependent effects, methodological differences in RPE assessment, or an absence of a meaningful perceptual benefit. GI discomfort could theoretically modify perceived exertion, but the included studies did not directly examine this relationship; it should therefore be regarded as an untested hypothesis rather than an explanation established by the present review.

GI tolerability also varied across studies. Four studies reported greater GI symptoms or discomfort with CAF + SB than with placebo, whereas five reported no statistically significant or substantial between-condition differences and three provided only descriptive findings. Moreover, some pronounced symptoms occurred specifically following sodium bicarbonate alone and therefore cannot be attributed to co-supplementation. Differences in the symptoms assessed, measurement scales, assessment time points, and reporting formats prevented consistent comparisons of symptom incidence, severity, duration, and effects on exercise performance. Accordingly, the available evidence does not support a uniform conclusion regarding the GI tolerability of CAF + SB. In practice, potential performance benefits should be considered alongside individual tolerance and factors such as dose, formulation, intake timing, meal conditions, and exercise characteristics [12,53]. Athletes considering CAF + SB should evaluate the supplementation strategy during training before its use in competition.

Several limitations should be acknowledged. First, relatively few studies were available for each quantitative outcome, limiting the precision of the pooled estimates and precluding reliable subgroup analyses, meta-regression, and formal assessment of publication bias or small-study effects. The consistently low I^2^ values for several outcomes should therefore not be interpreted as evidence of complete homogeneity, given the limited statistical power to detect heterogeneity and the differences in exercise protocols, supplementation strategies, participant characteristics, and outcome-assessment timing. These differences in exercise protocols also make the practical significance of the pooled mean-power difference uncertain, particularly when baseline power and task demands vary across studies. Second, 25 of the 29 study–outcome–comparison entries required paired variances to be estimated using an assumed within-participant correlation. Although alternative correlation assumptions generally produced similar effect directions and magnitudes, the statistical significance of the time-trial result varied across these assumptions. Furthermore, the mean-power and time-trial findings were sensitive to the exclusion of individual studies, indicating limited robustness. Consistently, the exploratory prediction intervals crossed the null value, indicating that the effects in future comparable exercise protocols may include no benefit. Third, all included studies were judged to raise some concerns regarding overall risk of bias. The low pre-consensus agreement in some RoB 2 domains, particularly D1, also indicates that incomplete reporting required subjective threshold judgments. Although disagreements were resolved through consensus using the RoB 2 algorithms, some uncertainty regarding the reproducibility of these classifications remains because of insufficient reporting of randomization procedures, potential period or carryover effects, and selective outcome reporting. Finally, heterogeneity and incomplete comparative testing of RPE and GI symptoms precluded quantitative synthesis and limited the interpretation of these outcomes. In addition, trial registries were not searched, and the restriction to English-language publications may have limited the identification of unpublished, ongoing, or non-English studies.

Future research should use adequately powered, preregistered four-condition crossover trials directly comparing placebo, caffeine, sodium bicarbonate, and CAF + SB, with prespecified interaction analyses to distinguish additional from synergistic effects. Such studies should adequately report randomization, counterbalancing, washout procedures, and paired variance information, while examining whether responses vary according to exercise intensity, duration, glycolytic demands, and supplementation dose and timing. Standardized assessment of performance, RPE, and GI symptoms is also needed to clarify efficacy and tolerability. Finally, integrating performance outcomes with direct measures of intramuscular acid–base status, neuromuscular function, and metabolic responses may help determine whether the physiological effects of CAF + SB contribute meaningfully to performance.

## 5. Conclusions

CAF + SB supplementation may produce modest improvements in selected exercise-performance outcomes compared with placebo. However, the evidence is based on a small number of heterogeneous crossover trials, and the pooled performance effects were sensitive to individual studies or analytical assumptions. CAF + SB did not consistently outperform caffeine or sodium bicarbonate alone; therefore, current evidence does not establish an additional ergogenic effect. Increases in blood bicarbonate and post-exercise lactate indicate physiological responses to supplementation but do not demonstrate superior performance. No consistent effect on RPE was observed, and gastrointestinal tolerability varied across studies. These findings should therefore be interpreted cautiously when considering the practical use of CAF + SB.

## Figures and Tables

**Figure 1:**
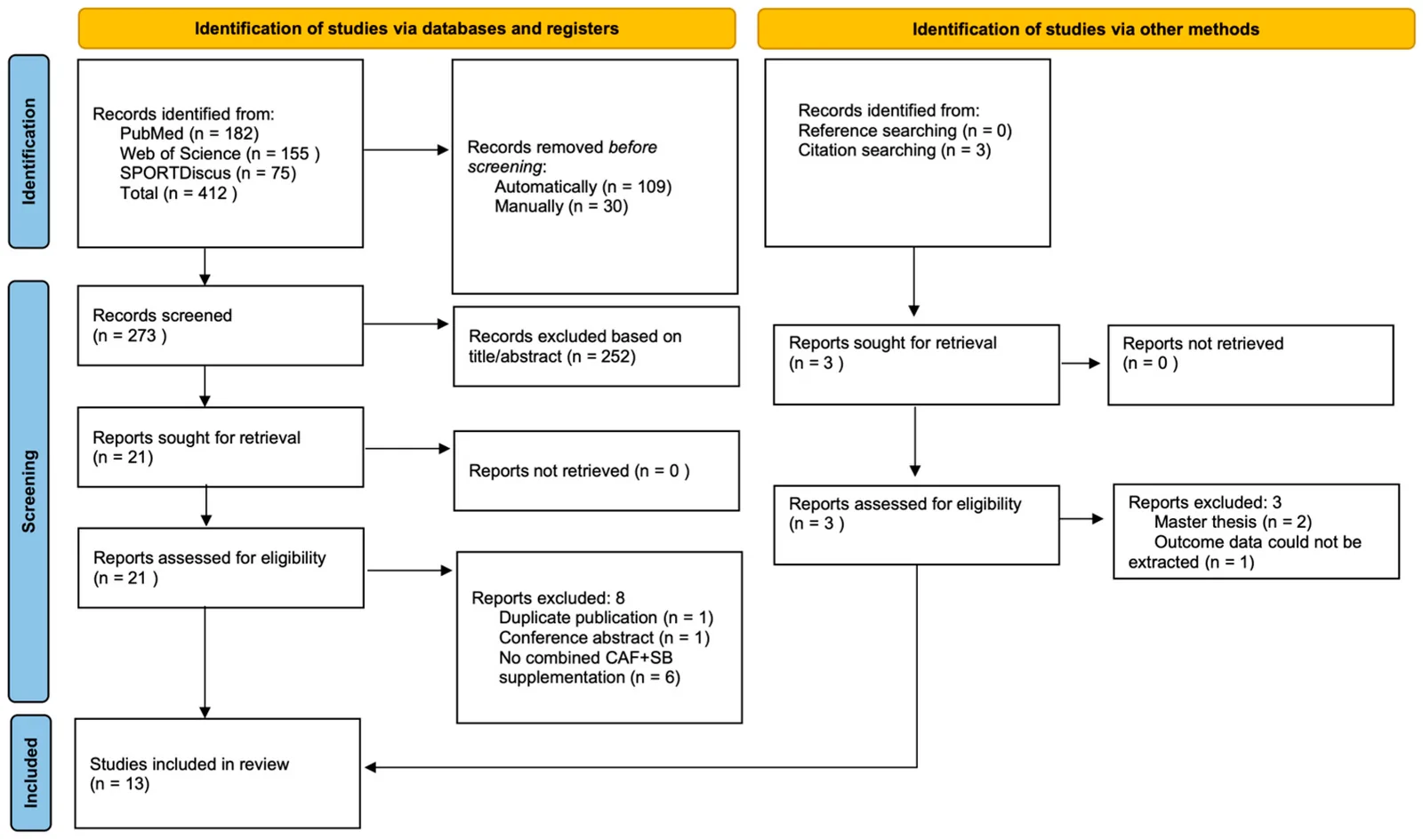
PRISMA flow diagram of the study selection process.

**Figure 2:**
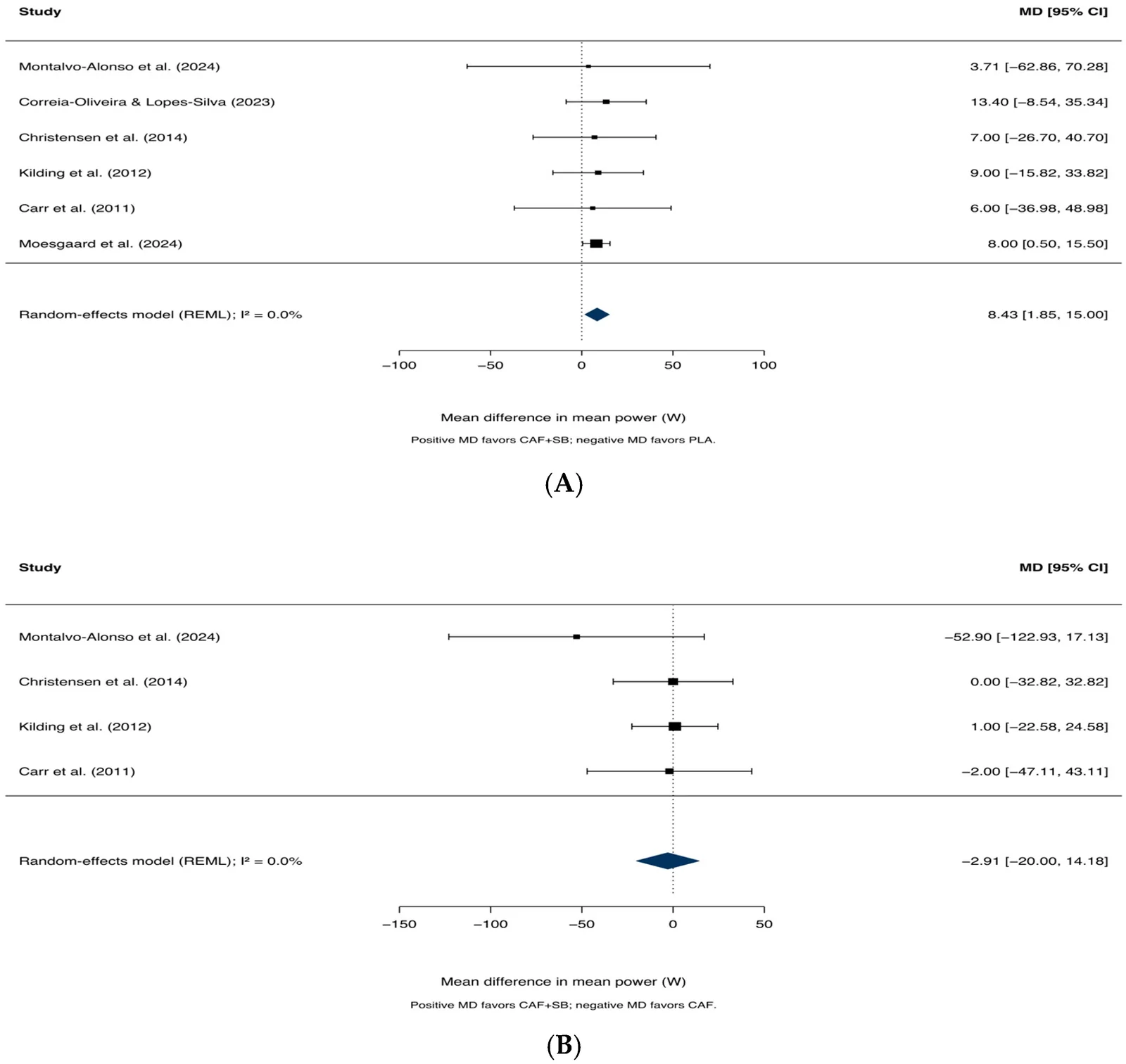

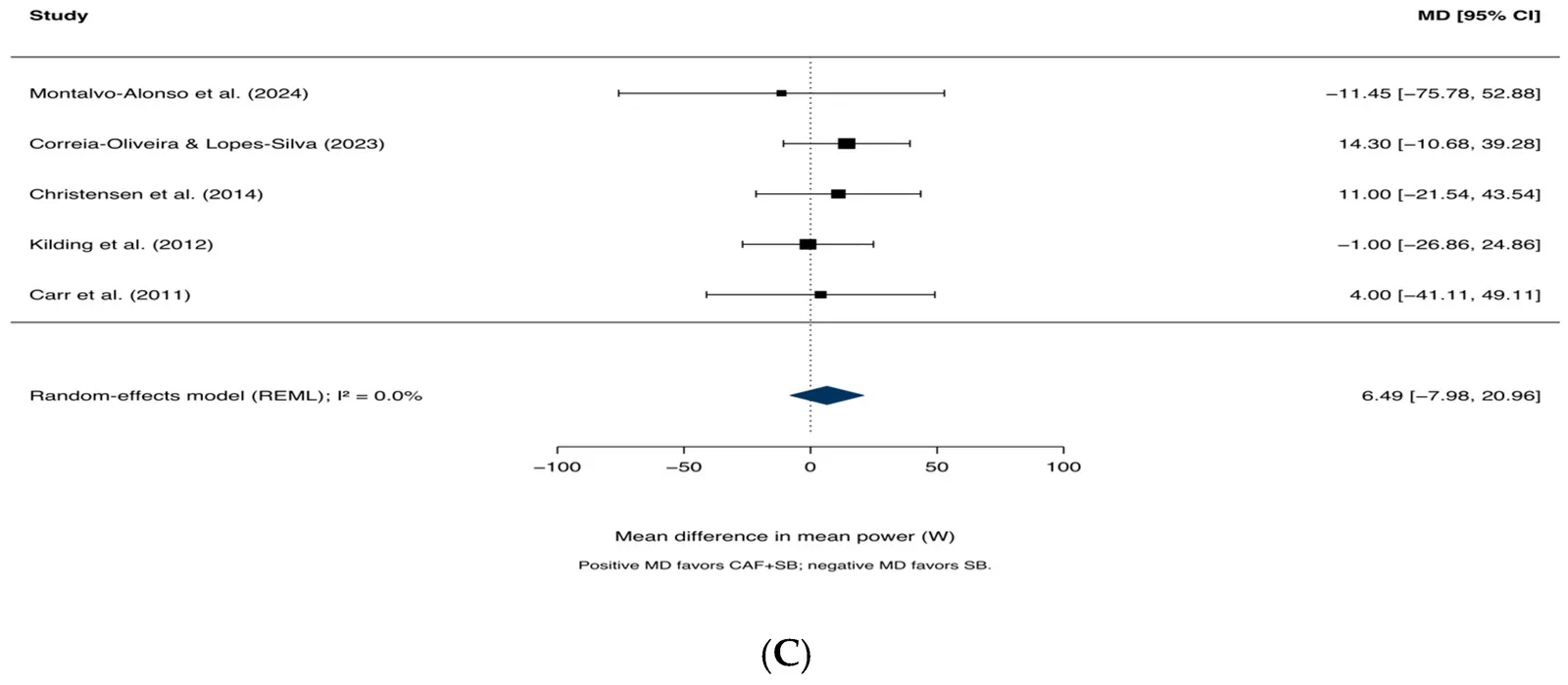
Forest plots of mean power. [20,21,35,36,38,39] (**A**) CAF + SB vs. placebo; (**B**) CAF + SB vs. caffeine alone; (**C**) CAF + SB vs. sodium bicarbonate alone. Squares represent the study-specific mean differences, with square size proportional to the study weight; horizontal lines indicate 95% CIs; diamonds represent the pooled random-effects estimates; and the vertical dashed line indicates the null effect (MD = 0). Note: For the estimate from Moesgaard et al. [39] in panel (**A**), the directly reported model-adjusted mean difference and 95% CI were used. Paired variances for all other estimates were derived from condition-specific SDs using an assumed within-participant correlation of r = 0.50. The significant pooled effect in panel (**A**) became non-significant after exclusion of Moesgaard et al [39]. Correlation sensitivity analyses using r = 0.30 and 0.70 were applied only to estimates requiring imputation. Detailed results are reported in Section 3.5 and Supplementary Files S4 and S5.

**Figure 3:**
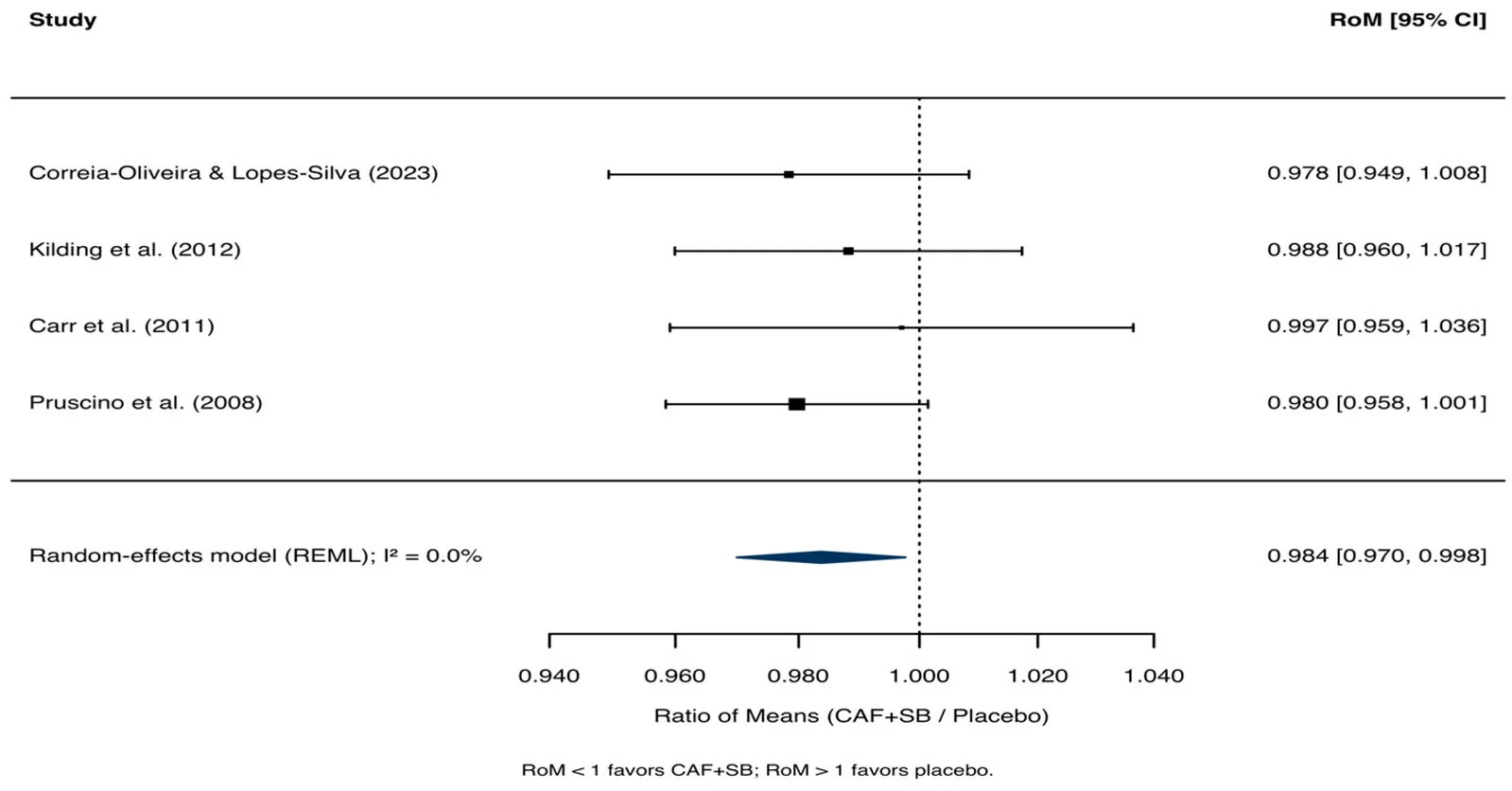
Forest plot of time-trial completion time [20,36,37,38]. CAF + SB vs. placebo. Squares represent the study-specific RoMs, with square size proportional to study weight; horizontal lines indicate 95% CIs; the diamond represents the pooled random-effects estimate; and the vertical dashed line indicates the null effect (RoM = 1). Note: Paired variances for all four studies were derived from condition-specific SDs using an assumed within-participant correlation of r = 0.50. The pooled effect became non-significant after exclusion of either Correia-Oliveira and Lopes-Silva [20] (RoM = 0.985, 95% CI = 0.970 to 1.001; *p* = 0.067) or Pruscino et al. [37] (RoM = 0.987, 95% CI = 0.969 to 1.005; *p* = 0.148), and when the assumed correlation was reduced to r = 0.30 (*p* = 0.052). Detailed sensitivity and influence analyses are reported in Section 3.5 and Supplementary Files S4 and S5.

**Figure 4:**
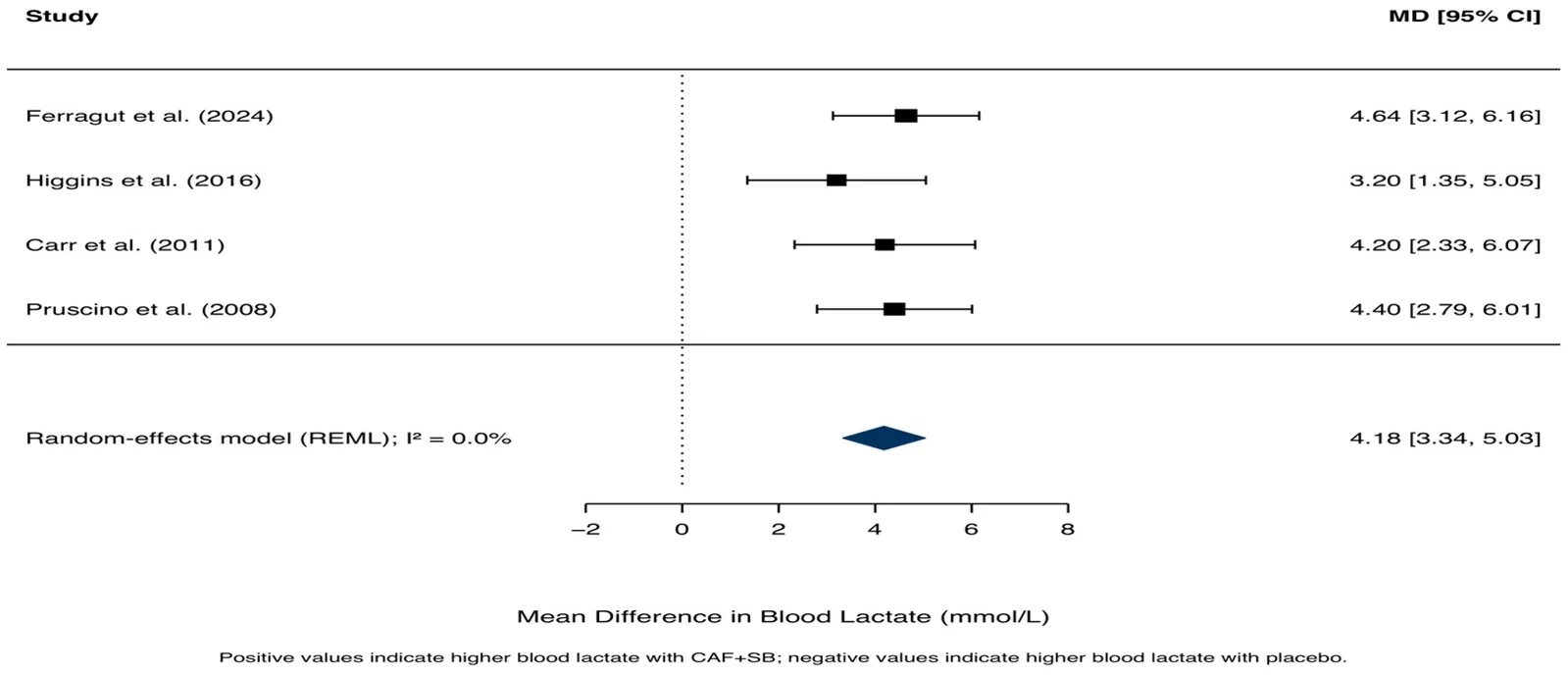
Forest plot of post-exercise blood lactate concentration [18,37,38,42]. CAF + SB vs. placebo. Squares represent the study-specific mean differences, with square size proportional to study weight; horizontal lines indicate 95% CIs; the diamond represents the pooled random-effects estimate; and the vertical dotted line indicates the null effect (MD = 0). Note: For Carr et al. [38], the directly reported SD of the paired difference was used. For the remaining three studies, paired variances were derived from condition-specific SDs using an assumed within-participant correlation of r = 0.50. Correlation sensitivity analyses using r = 0.30 and 0.70 were applied only to estimates requiring imputation. Detailed sensitivity analyses are reported in Section 3.5 and Supplementary Files S4 and S5.

**Figure 5:**
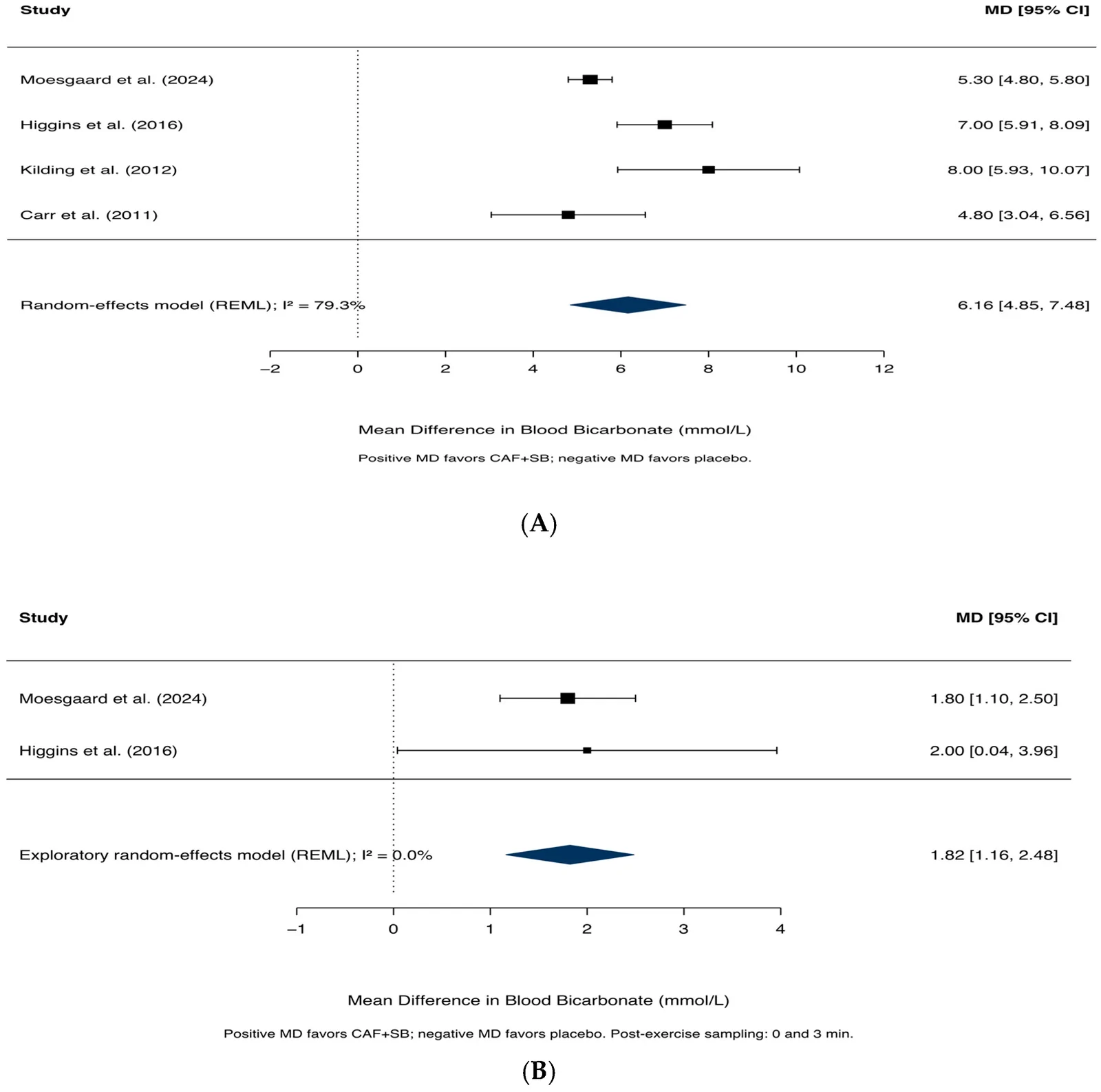
Forest plots of blood bicarbonate (HCO_3_^−^) concentrations [36,38,39,42]. (**A**) Pre-exercise HCO_3_^−^: CAF + SB vs. placebo; (**B**) Post-exercise HCO_3_^−^: CAF + SB vs. placebo. Squares represent the study-specific mean differences, with square size proportional to study weight; horizontal lines indicate 95% CIs; the diamond represents the pooled random-effects estimate; and the vertical dotted line indicates the null effect (MD = 0). Note: For Moesgaard et al. [39], the reported model-adjusted within-subject contrast and 95% CI were used in panel (**A**), and the reported mean difference and 95% CI were used in panel (**B**). For the remaining studies, paired variances were derived from condition-specific SDs using an assumed within-participant correlation of r = 0.50. Correlation sensitivity analyses using r = 0.30 and 0.70 were applied only to estimates requiring imputation. Detailed sensitivity analyses are reported in Section 3.5 and Supplementary Files S4 and S5.

**Table 1:** Characteristics of the included studies.

Study Characteristics		Participant Characteristics		Exercise Characteristics	Supplementation Regimen				Related Outcomes
Study	Country	Participant (*N*, Sex, Age, BM)	Training Status	Exercise Protocol	Washout	CAF (Dose, Timing)	SB (Dose, Timing)	Placebo/ Control	
Montalvo-Alonso et al. (2024) [21]	Spain	*N* = 28, 14 males/14 females Age = 23 ± 4 years BM = 68.2–68.5 kg	Recreationally trained (2.6 ± 1.2 years of training experience)	Bench press and back squat, 65% and 85% 1RM, one set to failure	≥72 h	3 mg/kg, 60 min pre-exercise	0.3 g/kg, 120 and 90 min pre-exercise (split into 2 doses)	Maltodextrin (volume matched)	Mean power, GI symptoms, RPE
Rezaei et al. (2019) [34]	Iran	*N* = 8, 8 males Age = 20.5 ± 2.4 years BM = 67.8 ± 7.7 kg	Elite karate athletes (>5 years of training experience, national level)	KSAT (incremental high intensity strikes to exhaustion)	7 days	6 mg/kg, 50 min pre-exercise	0.3 g/kg, 3-day loading, 120, 90, 60 min pre-exercise	Cellulose + CON	TTE, Blood lactate, RPE, GI symptoms
Correia-Oliveira and Lopes-Silva (2023) [20]	Brazil	*N* = 10, 10 males Age = 35.2 ± 7.3 years BM = 72.4 ± 6.7 kg VO_2_peak = 55.7 ± 7.9 mL/kg/min PPO = 306.4 ± 30.7 W	Recreationally trained cyclists (139.5 ± 46.9 min/day)	4-km cycling TT	3–7 days	5 mg/kg, 60 min pre-exercise	0.3 g/kg, 100 min pre-exercise	CaCO_3_ + cellulose	Mean power, TT, Blood lactate, Blood HCO_3_^−^, RPE, GI symptoms
Christensen et al. (2014) [35]	Denmark	*N* = 12, 11 males/1 female Age: 25 ± 1 years (open-weight males), 24 ± 3 years (light-weight males), 27 years (female) BW: 92 ± 3 kg (open-weight males), 75 ± 3 kg (light-weight males), 63 kg (female)	Elite rowers (international level)	6-min maximal rowing	NR (4 trials in 1 week)	3 mg/kg, 45 min pre-exercise	0.3 g/kg, 75 min pre-exercise	Dextrose + calcium	Mean power, Total distance, RPE, Stomach comfort
Ferragut et al. (2024) [18]	Spain	*N* = 25, 13 males/12 females Age = 23.3 ± 4.0 years BM = 69.6 ± 12.5 kg	Recreationally trained (3.1 ± 1.2 years, 4.5 ± 1.2 days/week)	4 × 30-s Wingate test (1.5-min passive rest)	≥72 h	3 mg/kg, 60 min pre-exercise	0.3 g/kg, 120 and 90 min (split into 2 doses) pre-exercise	Maltodextrin (matched volume)	Mean power, Peak power, Blood lactate, GI symptoms
Kilding et al. (2012) [36]	New Zealand	*N* = 10, 10 males Age = 24.2 ± 5.4 years BM = 79.1 ± 7.2 kg	Well-trained cyclists (9.1 ± 2.3 h/week)	3-km cycling TT	3 days	3 mg/kg, 60 min pre-exercise	0.3 g/kg, 120 and 90 min (serial) pre-exercise	Corn flour + matched capsules	TT, Mean power, Blood lactate, Blood HCO_3_^−^, RPE, GI symptoms
Pruscino et al. (2008) [37]	Australia	*N* = 6, 6 males Age: NR BM: NR	Highly trained elite male freestyle swimmers	2 × 200-m freestyle TT (30-min apart)	≥3 days	6.2 ± 0.3 mg/kg, 45 min pre-exercise	0.3 g/kg, 7 doses over 90 min pre-exercise	Glucose polymer	TT, Blood lactate, Blood HCO_3_^−^, Blood pH, GI symptoms
Carr et al. (2011) [38]	Australia	*N* = 8, 6 males/2 females Age: NR BM: 82.2 ± 12.2 kg (males), 77.5 ± 6.4 kg (females)	Well-trained rowers (national level)	2000-m rowing TT (Concept IID)	48 h	6 mg/kg, 30 min pre-exercise	0.3 g/kg, 90 min pre-exercise	Corn flour + glucose	Mean power, TT, Blood lactate, Blood HCO_3_^−^, RPE, GI symptoms
Kaçoğlu et al. (2024) [19]	Turkey	*N* = 19, 19 males Age = 23.6 ± 1.6 years BW = 64.3 ± 9.6 kg	Physically active male college students	Isometric Mid-Thigh Pull (IMTP)	7 days	6 mg/kg, 60 min pre-exercise	0.3 g/kg, 60 min pre-exercise	Maltodextrin	Peak IMTP force, Relative IMTP force, RPE
Felippe et al. (2016) [40]	Brazil	*N* = 10, 10 males Age: 23 ± 5 years BM: 66.0 ± 5.3 kg	Experienced judo athletes (15 ± 5 years practice, national level)	Special Judo Fitness Test (SJFT, 3 bouts)	7 days	6 mg/kg, 60 min pre-exercise	0.3 g/kg, 120, 90 min, 60 min pre-exercise	Cellulose	SJFT total throws, Blood lactate, RPE, GI symptoms
Moesgaard et al. (2024) [39]	Denmark	*N* = 23, 12 males/11 females Age: 25 ± 5 years (males), 25 ± 3 years (females) BM: NR VO_2_max: 56.7 ± 4.6 mL/kg/min (males), 50.2 ± 3.4 (females) mL/kg/min	Endurance-trained (>3 sessions/week, ≥2 years of training experience)	15-s sprint + 6-min cycling TT	>96 h	3 mg/kg, 60 min pre-exercise	0.3 g/kg, 60 min pre-exercise	Flavored water	6-min TT mean power, 15-s sprint power, MVC, Blood HCO_3_^−^, Blood pH, GI symptoms
Ziyaiyan et al. (2023) [41]	Iran	*N* = 20, 20 males Age: 22.30 ± 2.88 years BM: 80.6 ± 12.05 kg	CrossFit participants (≥2 years of training experience)	Cindy WOD (max rounds in 20 min)	7 days	6 mg/kg, 50 min pre-exercise	0.3 g/kg, 3-day loading, 120, 90, 60 min pre-exercise	Cellulose	Cindy performance, RPE, GI symptoms
Higgins et al. (2016) [42]	UK	*N* = 13, 13 males Age: 21 ± 3 years BM: 76 ± 12 kg Wpeak = 230 ± 34 W, VO_2_peak = 46 ± 8 mL/kg/min	Recreationally active (≥2 times/week, non-cyclists)	Cycling to volitional exhaustion at 100% Wpeak (TLIM)	≥48 h	5 mg/kg, 60 min pre-exercise	0.3 g/kg, 60 min pre-exercise	NaCl (taste-matched)	TTE, Blood lactate, Blood HCO_3_^−^, RPE, GI symptoms

Abbreviations: CAF = caffeine; SB = sodium bicarbonate; BM = body mass; BW = body weight; TT = time trial; TTE = time to exhaustion; GI = gastrointestinal; RPE = rating of perceived exertion; HCO_3_^−^ = bicarbonate; KSAT = Karate-Specific Aerobic Test; SJFT = Special Judo Fitness Test; IMTP = Isometric Mid-Thigh Pull; WOD = workout of the day; CON = no-supplement control; NR = not reported. Note: BM/BW terminology and training-status descriptors were retained as reported in the original studies and were not standardized into a common classification.

**Table 2:** Summary of rating of perceived exertion (RPE) in the included studies.

Study	RPE Scale and Assessment Time	Conditions Assessed	RPE Values as Reported	Reported Between-Condition Finding	Data Availability
Montalvo-Alonso et al. (2024) [21]	1–10 scale; after each trial	CAF + SB, CAF, SB, PLA	Numerical values not reported	No statistically significant between-condition difference (*p* > 0.05)	Inferential result only
Rezaei et al. (2019) [34]	Borg 6–20; after each KSAT level	CAF + SB, CAF, SB, PLA, CON	At level 7: CAF + SB, 15.37 ± 1.50; PLA, 17.50 ± 1.93; CON: 18.50 ± 0.76	Lower RPE with CAF + SB at levels 3–7 (*p* < 0.05); no statistically significant difference at the end of the KSAT (*p* = 0.11)	Selected numerical and inferential results
Correia-Oliveira and Lopes-Silva (2023) [20]	Borg 6–20; every 1 km during the time trial	CAF + SB, SB, PLA	Numerical values not reported	No statistically significant difference between CAF + SB and PLA (*p* = 0.74)	Inferential result only
Christensen et al. (2014) [35]	1–10 scale; after the 6-min performance test	CAF + SB, CAF, SB, PLA	CAF + SB, 8.8 ± 0.9; SB, 8.4 ± 0.9; CAF, 8.7 ± 1.4; PLA, 8.4 ± 1.2	No statistically significant treatment effect (*p* = 0.56)	Numerical and inferential results
Kilding et al. (2012) [36]	1–5 scale; timing not explicitly reported	CAF + SB, CAF, SB, PLA	CAF + SB, 4.0 ± 0.6; SB, 4.2 ± 0.8; CAF, 3.9 ± 0.8; PLA, 4.3 ± 0.7	Between-condition statistical comparison not reported	Numerical data only
Carr et al. (2011) [38]	Borg 6–20; immediately after the 2000-m test	CAF + SB, CAF, SB, PLA	CAF + SB, 17 ± 2; SB, 18 ± 2; CAF, 18 ± 2; PLA, 18 ± 2	CAF + SB was numerically 1 unit lower than PLA; between-condition statistical comparison not reported	Numerical data only
Kaçoğlu et al. (2024) [19]	Borg CR10; during the IMTP test	CAF + SB, CAF, SB, PLA, CON	RPE values not reported	Between-condition comparison not reported	Assessed; results not reported
Felippe et al. (2016) [40]	Borg 6–20 (15-point) scale; after each SJFT	CAF + SB, CAF, SB, PLA	SJFT1: CAF + SB, 10.0 ± 1.9; PLA, 10.0 ± 2.0. SJFT3: CAF + SB, 11.0 ± 2.4; PLA, 12.0 ± 2.9	No statistically significant treatment effect (*p* = 0.18); significant time effect (*p* = 0.009)	Numerical and inferential results
Ziyaiyan et al. (2023) [41]	1–10 scale; every 4 min and at the end of Cindy	CAF + SB, CAF, SB, PLA, CON	CAF + SB, 5.89 ± 0.42; SB, 6.41 ± 0.48; CAF, 6.06 ± 0.58; PLA, 6.37 ± 0.63; CON, 6.75 ± 0.48	Lower RPE with CAF + SB than with PLA (*p* = 0.001) and CON (*p* = 0.02)	Numerical and inferential results
Higgins et al. (2016) [42]	Borg 6–20 (local and overall RPE); at 1, 2, and 3 min during TLIM and at exhaustion	CAF + SB, CAF, SB, PLA	Local RPE at exhaustion: CAF + SB, 19 ± 1; SB, 20 ± 1; CAF, 20 ± 1; PLA, 20 ± 1	No statistically significant between-condition difference; CAF + SB was numerically approximately 1 unit lower at exhaustion	Numerical and inferential results; exact *p* value not reported

Abbreviations: CAF, caffeine; CAF + SB, caffeine combined with sodium bicarbonate; CON, control; IMTP, isometric mid-thigh pull; KSAT, Karate-Specific Aerobic Test; PLA, placebo; RPE, rating of perceived exertion; SB, sodium bicarbonate; SJFT, Special Judo Fitness Test; TLIM, time to volitional exhaustion. Note: Only studies that assessed RPE are shown. Numerical RPE values are presented as mean ± SD where available. “Inferential result only” indicates that a study reported a statistical comparison between conditions but did not report the corresponding numerical RPE values. When no statistical comparison was reported, no conclusion about between-condition differences was drawn.

**Table 3:** Summary of gastrointestinal symptoms in the included studies.

Study	Assessment Method and Timing	Conditions Assessed	GI Findings as Reported	Comparator-Specific Statistical Results	Data Availability
Montalvo-Alonso et al. (2024) [21]	Questionnaire; after each trial	CAF + SB, CAF, SB, PLA	CAF + SB: GI discomfort was 41% higher than with CAF and 52% higher than with PLA SB: GI discomfort was 39% higher than with CAF and 51% higher than with PLA	CAF + SB vs. CAF, *p* = 0.011 CAF + SB vs. PLA, *p* = 0.029 SB vs. CAF, *p* = 0.042 SB vs. PLA, *p* = 0.012	Numerical and inferential data
Rezaei et al. (2019) [34]	GI questionnaire; before and after the KSAT	CAF + SB, CAF, SB, PLA, CON	No severe GI discomfort was reported in any condition	Overall treatment effect: not statistically significant; exact *p* value unavailable. Comparator-specific pairwise results were not reported	Descriptive and inferential data
Correia-Oliveira and Lopes-Silva (2023) [20]	Validated side-effect questionnaire; timing not explicitly reported	CAF + SB, SB, PLA	One participant reported heartburn (5/10) after CAF + SB; no severe symptoms were reported	Overall between-condition effect: not statistically significant; exact *p* value unavailable. Comparator-specific pairwise results were not reported	Descriptive and inferential data
Christensen et al. (2014) [35]	Stomach comfort scale (1–10); before the 6-min performance test	CAF + SB, CAF, SB, PLA	CAF + SB, 2.4 ± 1.5; CAF, 1.8 ± 1.3; SB, 2.3 ± 1.2; PLA, 1.5 ± 0.7	Overall treatment effect: not statistically significant (*p* = 0.10). Comparator-specific pairwise results were not reported	Numerical and inferential data
Ferragut et al. (2024) [18]	Side-effect questionnaire; at the end of the trials	CAF + SB, CAF, SB, PLA	CAF + SB: GI discomfort was 45% higher than with CAF and 57% higher than with PLA SB: GI discomfort was 42% higher than with CAF and 54% higher than with PLA	CAF + SB vs. CAF, *p* = 0.005 CAF + SB vs. PLA, *p* = 0.021 SB vs. CAF, *p* = 0.039 SB vs. PLA, *p* = 0.012	Numerical and inferential data
Kilding et al. (2012 [36])	Gastric discomfort rating (1–5); timing not explicitly reported	CAF + SB, CAF, SB, PLA	CAF + SB, 1.55 ± 0.60; CAF, 1.40 ± 0.48; SB, 1.40 ± 0.52; PLA, 1.35 ± 0.60	Comparator-specific inferential comparison not reported	Numerical and inferential data
Pruscino et al. (2008) [37]	Questionnaire; the entire testing period	CAF + SB, CAF, SB, PLA	Some participants reported stomach cramps, diarrhea, or dizziness after bicarbonate- or caffeine-containing conditions	Comparator-specific statistical comparison not reported	Descriptive data only
Carr et al. (2011) [38]	Posttest questionnaire; after the 2000-m test	CAF + SB, CAF, SB, PLA	All participants reported GI side effects (including nausea, vomiting, and stomach pain) after SB	Comparator-specific statistical comparison not reported	Descriptive data only
Moesgaard et al. (2024) [39]	Side-effect reporting; throughout each trial	CAF + SB, CAF, SB, PLA	Participants reporting side effects: CAF + SB, 12; CAF, 1; SB, 8; PLA, 2. Reported symptoms included stomach discomfort (n = 11), nausea (n = 8), diarrhea (n = 9), and dizziness (n = 2)	Comparator-specific statistical comparison not reported	Descriptive data only
Felippe et al. (2016) [40]	GI questionnaire; before and after the SJFT	CAF + SB, CAF, SB, PLA	After SB, two participants reported flatulence (scores 6 and 9) and one reported an urge to defecate (score 7); after CAF+SB, one participant reported flatulence (score 10)	Overall between-condition effect: not statistically significant; exact *p* value unavailable. Comparator-specific pairwise results were not reported	Descriptive and inferential data
Ziyaiyan et al. (2023) [41]	GI questionnaire (0–9); during the study	CAF + SB, CAF, SB, PLA, CON	Minor heartburn and mild diarrhea were reported	CAF + SB vs. PLA: significantly greater GI side effects; exact *p* value unavailable SB vs. PLA: significantly greater GI side effects; exact *p* value unavailable	Descriptive and inferential data
Higgins et al. (2016) [42]	Abdominal discomfort and gastrointestinal fullness ratings; before ingestion, 30 min, 60 min, and at the end of exercise	CAF + SB, CAF, SB, PLA	Abdominal discomfort was greater with CAF + SB and SB than with PLA before exercise (3 ± 2 vs. 1 ± 2) and at the end of exercise (3 ± 3 vs. 1 ± 2)	CAF + SB vs. PLA, *p* < 0.01 SB vs. PLA, *p* < 0.01	Numerical and inferential data

Abbreviations: CAF, caffeine; CAF + SB, caffeine combined with sodium bicarbonate; CON, control; GI, gastrointestinal; KSAT, Karate-Specific Aerobic Test; PLA, placebo; SB, sodium bicarbonate; SJFT, Special Judo Fitness Test. Note: Only studies that assessed GI symptoms are included. Only explicitly reported pairwise statistical results are listed. The inclusion of a condition in a study does not indicate that a comparator-specific statistical result was available for that contrast. Findings involving SB alone are reported separately and were not attributed to CAF+SB. Overall treatment effects and descriptive symptom data were not interpreted as comparator-specific differences. Values are presented as mean ± SD, counts, symptom scores, or reported between-condition differences.

## Data Availability

No new primary data were collected. Data for this meta-analysis were extracted from the published studies cited in the manuscript. Study- and outcome-specific variance sources and within-participant correlation assumptions are provided in Supplementary File S2; sensitivity-analysis results are provided in Supplementary Files S4 and S5. The consolidated extracted dataset and analysis code are not publicly available.

## Supplementary Materials

The following supporting information can be downloaded at: https://www.mdpi.com/article/10.3390/nu18193171/s1, Supplementary File S1: Search strategy; Supplementary File S2: Study- and outcome-specific sources of paired variance estimates and within-participant correlation assumptions; Supplementary File S3: Risk of bias assessment; Supplementary File S4: Leave-one-out sensitivity and influence analyses; Supplementary File S5: Correlation sensitivity analyses; Supplementary File S6: GRADE certainty of evidence; Supplementary File S7: PRISMA 2020 checklist.
