# Supplementary material for: The Effects of Combined Caffeine and Sodium Bicarbonate Supplementation on Exercise Performance, Physiological Responses, and Gastrointestinal Tolerability: A Systematic Review and Meta-Analysis
Source: Nutrients. 2026 Sep 26;18(19):3171. doi: 10.3390/nu18193171 (PMC13666935; doi:10.3390/nu18193171)
Supplement: Supplementary file 1 [file nutrients-18-03171-s001.zip › Supplementary File S1 Search strategy.pdf]

Table S1. Complete search strategy for all databases.

Databases	Exact search date	Search Strategy	Results
PubMed	17 July 2026	("Caffeine"[Mesh] OR caffeine[tiab] OR caffeinated[tiab] OR CAF[tiab]) AND ("Sodium Bicarbonate"[Mesh] OR "sodium bicarbonate"[tiab] OR bicarbonate[tiab] OR NaHCO3[tiab] OR "sodium hydrogen carbonate"[tiab])	182
Web of Science Core Collection	17 July 2026	TS=((caffeine OR caffeinated OR CAF) AND ("sodium bicarbonate" OR bicarbonate OR NaHCO3 OR "sodium hydrogen carbonate"))	155
SPORTDiscus	18 July 2026	AB ((caffeine OR caffeinated)) AND AB (("sodium bicarbonate" OR bicarbonate OR NaHCO3 OR "sodium hydrogen carbonate"))	75
Overall			412
