# Supplementary material for: The Effects of Combined Caffeine and Sodium Bicarbonate Supplementation on Exercise Performance, Physiological Responses, and Gastrointestinal Tolerability: A Systematic Review and Meta-Analysis
Source: Nutrients. 2026 Sep 26;18(19):3171. doi: 10.3390/nu18193171 (PMC13666935; doi:10.3390/nu18193171)
Supplement: Supplementary file 1 [file nutrients-18-03171-s001.zip › Supplementary File S3 Risk of bias assessment.pdf]

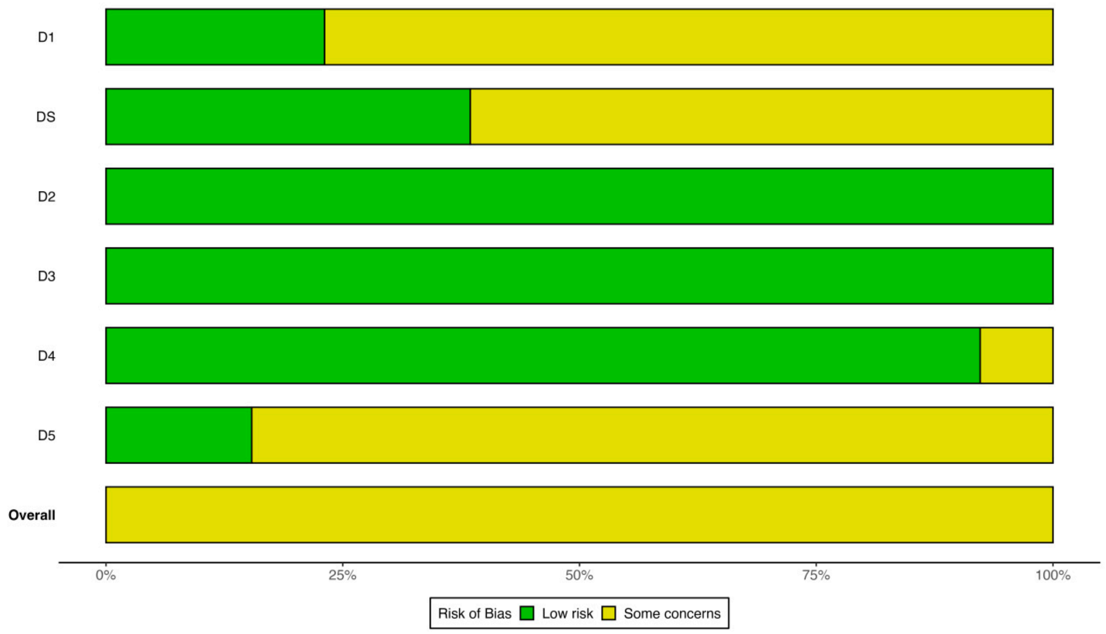

Figure S3-1. Summary plot of risk-of-bias judgements for included studies.

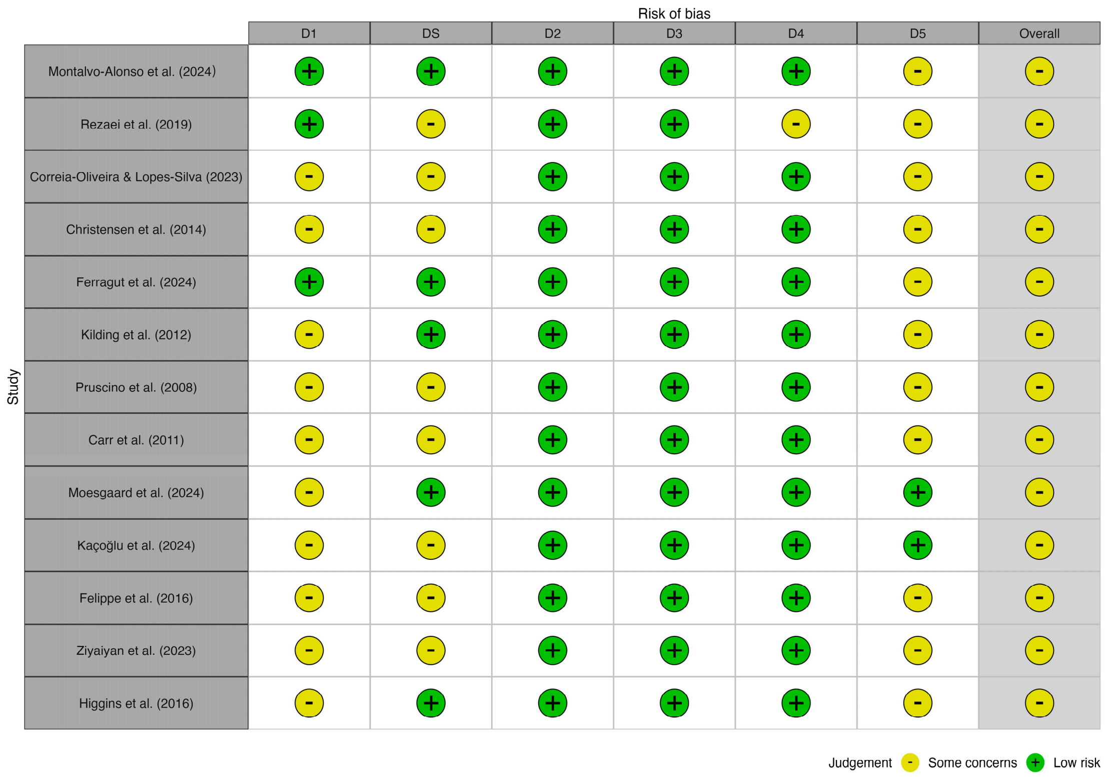

Figure S3-2. Risk-of-bias domain-level judgements for individual studies.

Note: D1, bias arising from the randomization process; DS, bias arising from period and carryover effects; D2, bias due to deviations from intended interventions; D3, bias due to missing outcome data; D4, bias in measurement of the outcome; D5, bias in selection of the reported result. Green symbols indicate low risk of bias, yellow symbols indicate some concerns.

Table S3-1. Signalling-question assessments and consensus judgements for Domain S.

Study	S.1	S.2	S.3	Supporting information	Final judgement	Consensus rationale
Montalvo-Alonso et al. (2024)	PY	NA	Y	Randomized, double-blind, placebo-controlled crossover; four conditions (SB+CAF, SB, CAF, PLA); sequence counterbalanced using www.randomizer.org; washout ≥72 h between sessions.	Low risk	The allocation to intervention sequences was adequately reported and counterbalanced (S.1 = PY), and the washout interval of at least 72 h was considered sufficient to eliminate carryover effects (S.3 = Y). Period effects were not explicitly tested, but this was not applicable because sequence allocation was balanced (S.2 = NA). Low risk of bias was judged for Domain S.
Rezaei et al. (2019)	NI	NI	Y	Double-blind, crossover, randomized placebo-controlled; five conditions (CON, PLA, CAF, SB, CAF+SB); washout period 7 days; same time of day for all assessments.	Some concerns	The allocation to intervention sequences was not adequately reported (S.1 = NI), and it was unclear whether period effects were accounted for in the analysis (S.2 = NI). Although the reported washout interval of 7 days was considered sufficient (S.3 = Y), some concerns remained regarding potential period effects.
Correia-Oliveira and Lopes-Silva (2023)	NI	NI	Y	Double-blind, counterbalanced, repeated-measures crossover; three conditions (CAF+SB, SB, PLA); 3- to 7-day interval for washing out; same time of day.	Some concerns	The allocation to intervention sequences was not adequately reported (S.1 = NI), and period effects were not clearly accounted for in the analysis (S.2 = NI). Although the reported washout interval of 3–7 days was considered sufficient (S.3 = Y), some concerns remained regarding potential period effects.
Christensen et al. (2014)	NI	PN	PN	Double-blind, randomized, placebo-controlled; four conditions (CAF, SB, CAF+SB, PLA); four trials on separate days within a week; caffeine avoided 36 h before testing.	Some concerns	The allocation to intervention sequences was not adequately reported (S.1 = NI), and period effects were probably not accounted for in the analysis (S.2 = PN). The washout interval was probably not sufficient, as four trials were performed within a single week (approximately 24–48 h between trials), which may be too short to fully eliminate carryover effects (S.3 = PN). Some concerns were judged for Domain S.
Ferragut et al. (2024)	PY	NA	Y	Randomized, double-blind, placebo-controlled crossover; four conditions (CAF+SB, SB, CAF, PLA); washout ≥72 h; sequence randomized via www.randomized.org.	Low risk	The allocation to intervention sequences was adequately reported and randomized (S.1 = PY), and the washout interval of at least 72 h was considered sufficient (S.3 = Y). Period effects were not applicable beca

Study	S.1	S.2	S.3	Supporting information	Final judgement	Consensus rationale
						use sequence allocation was balanced (S.2 = NA). L ow risk of bias was judged for Domain S.
Kilding et al. (2012)	PY	NA	Y	Double-blind, crossover; four conditions (CAF, SB, CAF+SB, PLA); balanced ran dom order; trials on Days 4, 7, 10, 13 (approximately 3 days apart).	Low risk	The allocation to intervention sequences was adequate ly reported and balanced (S.1 = PY), and the washo ut interval of approximately 3 days was considered s ufficient (S.3 = Y). Period effects were not applicabl e because sequence allocation was balanced (S.2 = N A). Low risk of bias was judged for Domain S.
Pruscino et al. (2008)	NI	NI	Y	Double-blind, randomized crossover; fourc onditions (SB, CAF, CAF+SB, PLA); fou r testing days separated by at least 3 da ys; caffeine abstained 48 h before each t rial.	Some concerns	The allocation to intervention sequences was not ade quately reported (S.1 = NI), and period effects were not clearly accounted for in the analysis (S.2 = NI). Although the reported washout interval of at least 3 days was considered sufficient (S.3 = Y), some conc erns remained regarding potential period effects.
Carr et al. (2011)	NI	NI	PY	Double-blind, crossover, semicounterbalan ced; four conditions (SB, CAF, CAF+SB, PLA); 48 h between trials to replicate r egatta timing; two cohorts.	Some concerns	The allocation to intervention sequences was not ade quately reported (S.1 = NI), and period effects were not clearly accounted for in the analysis (S.2 = NI). The washout interval of 48 h was probably sufficient for caffeine and SB clearance, but it was chosen to simulate race scheduling rather than for washout pur poses, so it was judged as probably yes (S.3 = PY). Some concerns remained regarding period effects.
Moesgaard et al. (2024)	PY	NA	Y	Double-blind, randomized, placebo-control led; four experimental trials (CAF, SB, C AF+SB, PLA) in randomized order-balanc ed manner; >96 h between trials.	Low risk	The allocation to intervention sequences was adequate ly reported and balanced (S.1 = PY), and the washo ut interval of more than 96 h was considered sufficie nt (S.3 = Y). Period effects were not applicable beca use sequence allocation was balanced (S.2 = NA). L ow risk of bias was judged for Domain S.
Kaçoğlu et al. (2024)	NI	NI	Y	Crossover, double-blind, placebo-controlle d; five conditions (CON, PLA, CAF, SB, CAF+SB); washout ≥7 days; same day of week and time of day.	Some concerns	The allocation to intervention sequences was not ade quately reported (S.1 = NI), and period effects were not clearly accounted for in the analysis (S.2 = NI). Although the reported washout interval of at least 7 days was considered sufficient (S.3 = Y), some conc erns remained regarding potential period effects.

Study	S.1	S.2	S.3	Supporting information	Final judgement	Consensus rationale
Felippe et al. (2016)	NI	NI	Y	Double-blind, randomized, counterbalanced, partial Latin-square; four conditions (PLA, SB, CAF, CAF+SB); 1-week washout between conditions.	Some concerns	The allocation to intervention sequences was not adequately reported (S.1 = NI), and period effects were not clearly accounted for in the analysis (S.2 = NI). Although the reported washout interval of 1 week was considered sufficient (S.3 = Y), some concerns remained regarding potential period effects.
Ziyaiyan et al. (2023)	NI	NI	Y	Double-blind, crossover, randomized, placebo-controlled; five conditions (CON, PLA, CAF, SB, CAF+SB); 7-day washout; same time of day.	Some concerns	The allocation to intervention sequences was not adequately reported (S.1 = NI), and period effects were not clearly accounted for in the analysis (S.2 = NI). Although the reported washout interval of 7 days was considered sufficient (S.3 = Y), some concerns remained regarding potential period effects.
Higgins et al. (2016)	PY	NA	Y	Counterbalanced, crossover, double-blind; four conditions (SB, CAF, CAF+SB, PLA); each visit separated by at least 48 h.	Low risk	The allocation to intervention sequences was adequately reported and counterbalanced (S.1 = PY), and the washout interval of at least 48 h was considered sufficient (S.3 = Y). Period effects were not applicable because sequence allocation was balanced (S.2 = NA). Low risk of bias was judged for Domain S.

Note: S.1, balance in the number of participants allocated to each intervention sequence; S.2, consideration of period effects in the analysis when sequence allocation was not balanced or was insufficiently reported; S.3, sufficiency of the interval for potential carryover effects to have disappeared before outcome assessment in the subsequent period. Y, yes; PY, probably yes; N, no; PN, probably no; NI, no information; NA, not applicable.

Table S3-2. Inter-rater agreement for risk-of-bias assessments.

Domain	N	Agreement, n	Agreement, %	Cohen's κ
D1	13	3	23.1%	−0.121
DS	13	9	69.2%	0.350
D2	13	13	100.0%	Not estimable
D3	13	12	92.3%	0.000
D4	13	10	76.9%	−0.114
D5	13	9	69.2%	0.161
Overall	13	7	53.8%	0.000

Note: The low or negative kappa values indicated substantial differences between the reviewers' initial independent judgements in several domains. These disagreements primarily concerned the interpretation of insufficiently reported randomization procedures and the application of judgement thresholds. Consensus judgements were reached by re-examining the original study reports and applying the relevant RoB 2 signalling questions and decision algorithms.

Table S3-3. Independent pre-consensus and final consensus risk-of-bias judgements.

Study	Domain	Reviewer 1	Reviewer 2	Agreement	Final consensus
Montalvo-Alonso et al. (2024)	D1	Low	Low	Agree	Low
Montalvo-Alonso et al. (2024)	DS	Low	Some concerns	Disagree	Low
Montalvo-Alonso et al. (2024)	D2	Low	Low	Agree	Low
Montalvo-Alonso et al. (2024)	D3	Low	Low	Agree	Low
Montalvo-Alonso et al. (2024)	D4	Low	Low	Agree	Low
Montalvo-Alonso et al. (2024)	D5	Some concerns	Some concerns	Agree	Some concerns
Montalvo-Alonso et al. (2024)	Overall	Some concerns	Some concerns	Agree	Some concerns
Rezaei et al. (2019)	D1	Low	Low	Agree	Low
Rezaei et al. (2019)	DS	Some concerns	Some concerns	Agree	Some concerns
Rezaei et al. (2019)	D2	Low	Low	Agree	Low
Rezaei et al. (2019)	D3	Low	Some concerns	Disagree	Low
Rezaei et al. (2019)	D4	Some concerns	Low	Disagree	Some concerns
Rezaei et al. (2019)	D5	Some concerns	Some concerns	Agree	Some concerns
Rezaei et al. (2019)	Overall	Some concerns	Some concerns	Agree	Some concerns
Correia-Oliveira and Lopes-Silva (2023)	D1	Some concerns	Low	Disagree	Some concerns
Correia-Oliveira and Lopes-Silva (2023)	DS	Some concerns	Some concerns	Agree	Some concerns
Correia-Oliveira and Lopes-Silva (2023)	D2	Low	Low	Agree	Low
Correia-Oliveira and Lopes-Silva (2023)	D3	Low	Low	Agree	Low
Correia-Oliveira and Lopes-Silva (2023)	D4	Low	Low	Agree	Low
Correia-Oliveira and Lopes-Silva (2023)	D5	Some concerns	Low	Disagree	Some concerns
Correia-Oliveira and Lopes-Silva (2023)	Overall	Some concerns	Some concerns	Agree	Some concerns
Christensen et al. (2014)	D1	Some concerns	Low	Disagree	Some concerns
Christensen et al. (2014)	DS	Some concerns	Some concerns	Agree	Some concerns
Christensen et al. (2014)	D2	Low	Low	Agree	Low
Christensen et al. (2014)	D3	Low	Low	Agree	Low
Christensen et al. (2014)	D4	Low	Some concerns	Disagree	Low
Christensen et al. (2014)	D5	Some concerns	Some concerns	Agree	Some concerns
Christensen et al. (2014)	Overall	Some concerns	Some concerns	Agree	Some concerns
Ferragut et al. (2024)	D1	Low	Some concerns	Disagree	Low
Ferragut et al. (2024)	DS	Low	Low	Agree	Low

Study	Domain	Reviewer 1	Reviewer 2	Agreement	Final consensus
Ferragut et al. (2024)	D2	Low	Low	Agree	Low
Ferragut et al. (2024)	D3	Low	Low	Agree	Low
Ferragut et al. (2024)	D4	Low	Low	Agree	Low
Ferragut et al. (2024)	D5	Some concerns	Some concerns	Agree	Some concerns
Ferragut et al. (2024)	Overall	Some concerns	Some concerns	Agree	Some concerns
Kilding et al. (2012)	D1	Some concerns	Low	Disagree	Some concerns
Kilding et al. (2012)	DS	Low	Some concerns	Disagree	Low
Kilding et al. (2012)	D2	Low	Low	Agree	Low
Kilding et al. (2012)	D3	Low	Low	Agree	Low
Kilding et al. (2012)	D4	Low	Low	Agree	Low
Kilding et al. (2012)	D5	Some concerns	Some concerns	Agree	Some concerns
Kilding et al. (2012)	Overall	Some concerns	Some concerns	Agree	Some concerns
Pruscino et al. (2008)	D1	Some concerns	Low	Disagree	Some concerns
Pruscino et al. (2008)	DS	Some concerns	Low	Disagree	Some concerns
Pruscino et al. (2008)	D2	Low	Low	Agree	Low
Pruscino et al. (2008)	D3	Low	Low	Agree	Low
Pruscino et al. (2008)	D4	Low	Low	Agree	Low
Pruscino et al. (2008)	D5	Some concerns	Some concerns	Agree	Some concerns
Pruscino et al. (2008)	Overall	Some concerns	High	Disagree	Some concerns
Carr et al. (2011)	D1	Some concerns	Some concerns	Agree	Some concerns
Carr et al. (2011)	DS	Some concerns	Low	Disagree	Some concerns
Carr et al. (2011)	D2	Low	Low	Agree	Low
Carr et al. (2011)	D3	Low	Low	Agree	Low
Carr et al. (2011)	D4	Low	Low	Agree	Low
Carr et al. (2011)	D5	Some concerns	Low	Disagree	Some concerns
Carr et al. (2011)	Overall	Some concerns	Low	Disagree	Some concerns
Kaçoğlu et al. (2024)	D1	Some concerns	Low	Disagree	Some concerns
Kaçoğlu et al. (2024)	DS	Some concerns	Some concerns	Agree	Some concerns
Kaçoğlu et al. (2024)	D2	Low	Low	Agree	Low
Kaçoğlu et al. (2024)	D3	Low	Low	Agree	Low
Kaçoğlu et al. (2024)	D4	Low	Low	Agree	Low
Kaçoğlu et al. (2024)	D5	Low	Some concerns	Disagree	Low
Kaçoğlu et al. (2024)	Overall	Some concerns	Low	Disagree	Some concerns

Study	Domain	Reviewer 1	Reviewer 2	Agreement	Final consensus
Felippe et al. (2016)	D1	Some concerns	Low	Disagree	Some concerns
Felippe et al. (2016)	DS	Some concerns	Some concerns	Agree	Some concerns
Felippe et al. (2016)	D2	Low	Low	Agree	Low
Felippe et al. (2016)	D3	Low	Low	Agree	Low
Felippe et al. (2016)	D4	Low	Some concerns	Disagree	Low
Felippe et al. (2016)	D5	Some concerns	Some concerns	Agree	Some concerns
Felippe et al. (2016)	Overall	Some concerns	Low	Disagree	Some concerns
Moesgaard et al. (2024)	D1	Some concerns	Low	Disagree	Some concerns
Moesgaard et al. (2024)	DS	Low	Low	Agree	Low
Moesgaard et al. (2024)	D2	Low	Low	Agree	Low
Moesgaard et al. (2024)	D3	Low	Low	Agree	Low
Moesgaard et al. (2024)	D4	Low	Low	Agree	Low
Moesgaard et al. (2024)	D5	Low	Low	Agree	Low
Moesgaard et al. (2024)	Overall	Some concerns	Low	Disagree	Some concerns
Ziyaiyan et al. (2023)	D1	Some concerns	Low	Disagree	Some concerns
Ziyaiyan et al. (2023)	DS	Some concerns	Some concerns	Agree	Some concerns
Ziyaiyan et al. (2023)	D2	Low	Low	Agree	Low
Ziyaiyan et al. (2023)	D3	Low	Low	Agree	Low
Ziyaiyan et al. (2023)	D4	Low	Low	Agree	Low
Ziyaiyan et al. (2023)	D5	Some concerns	Some concerns	Agree	Some concerns
Ziyaiyan et al. (2023)	Overall	Some concerns	Some concerns	Agree	Some concerns
Higgins et al. (2016)	D1	Some concerns	Low	Disagree	Some concerns
Higgins et al. (2016)	DS	Low	Low	Agree	Low
Higgins et al. (2016)	D2	Low	Low	Agree	Low
Higgins et al. (2016)	D3	Low	Low	Agree	Low
Higgins et al. (2016)	D4	Low	Low	Agree	Low
Higgins et al. (2016)	D5	Some concerns	Low	Disagree	Some concerns
Higgins et al. (2016)	Overall	Some concerns	Low	Disagree	Some concerns

Note: Reviewer 1 and Reviewer 2 independently assessed each study before consensus. Agreement indicates whether their initial domain-level judgements were identical. Final consensus judgements were established after the reviewers re-examined the original study reports and applied the relevant RoB 2 signalling questions and decision algorithms. D1, bias arising from the randomization process; DS, bias arising from period and carryover effects; D2, bias due to deviations from intended interventions; D3, bias due to missing outcome data; D4, bias in measurement of the outcome; D5, bias in selection of the reported result.
