# Supplementary material for: The Effects of Combined Caffeine and Sodium Bicarbonate Supplementation on Exercise Performance, Physiological Responses, and Gastrointestinal Tolerability: A Systematic Review and Meta-Analysis
Source: Nutrients. 2026 Sep 26;18(19):3171. doi: 10.3390/nu18193171 (PMC13666935; doi:10.3390/nu18193171)
Supplement: Supplementary file 1 [file nutrients-18-03171-s001.zip › Supplementary File S6 GRADE certainty of evidence.pdf]

Supplementary Table S6. GRADE assessment of the certainty of evidence

Panel A. CAF+SB versus placebo

Outcome	Studies (participants)	Effect estimate or narrative finding	Risk of bias	Inconsistency	Indirectness	Imprecision	Publication bias	Certainty	Importance
Mean power	6 (91)	MD 8.43 W higher (95% CI 1.85 to 15.00 higher)	Serious[a]	Not serious	Not serious	Serious[b]	Undetected[j]	Low ⊕⊕◯◯	Critical
Time-trial completion time	4 (34)	RoM 0.984 (95% CI 0.970 to 0.998), corresponding to 1.6% shorter completion time (0.2% to 3.0% shorter)	Serious[a]	Not serious	Not serious	Serious[c]	Undetected[j]	Low ⊕⊕◯◯	Critical
Blood lactate concentration	4 (52)	MD 4.18 mmol/L higher (95% CI 3.34 to 5.03 higher)	Serious[a]	Not serious	Not serious	Not serious	Undetected[j]	Moderate ⊕⊕⊕◯	Important
Pre-exercise blood bicarbonate concentration	4 (54)	MD 6.16 mmol/L higher (95% CI 4.85 to 7.48 higher)	Serious[a]	Serious[d]	Not serious	Not serious	Undetected[j]	Low ⊕⊕◯◯	Important
Post-exercise blood bicarbonate concentration	2 (36)	MD 1.82 mmol/L higher (95% CI 1.16 to 2.48 higher)	Serious[a]	Not serious	Not serious	Serious[e]	Undetected[j]	Low ⊕⊕◯◯	Important
Rating of perceived exertion	9 (119)	Two studies reported lower RPE under CAF+SB at selected assessment points, five reported no treatment or between-condition effect, and two reported condition-specific values without formal comparison; results were not pooled.	Serious[a]	Serious[f]	Not serious	Serious[g]	Undetected[j]	Very low ⊕◯◯◯	Important
Gastrointestinal symptoms	10 (159)	Four studies reported greater GI symptoms or discomfort with CAF+SB than with placebo. Five studies reported no statistically significant overall treatment or between-condition effect, although comparator-specific pairwise results were not consistently available. One study provided descriptive counts without a formal between-condition comparison; results were not pooled.	Serious[a]	Serious[h]	Not serious	Serious[i]	Undetected[j]	Very low ⊕◯◯◯	Important

Panel B. CAF+SB versus caffeine alone

Outcome	Studies (participants)	Effect estimate or narrative finding	Risk of bias	Inconsistency	Indirectness	Imprecision	Publication bias	Certainty	Importance
Mean power	4 (58)	MD -2.91 W lower (95% CI -20.00 lower to 14.18 higher)	Serious[a]	Not serious	Not serious	Serious[k]	Undetected[j]	Low ⊕⊕◯◯	Critical

Panel C. CAF+SB versus sodium bicarbonate alone

Outcome	Studies (participants)	Effect estimate or narrative finding	Risk of bias	Inconsistency	Indirectness	Imprecision	Publication bias	Certainty	Importance
Mean power	5 (68)	MD 6.49 W higher (95% CI -7.98 lower to 20.96 higher)	Serious[a]	Not serious	Not serious	Serious[l]	Undetected[j]	Low ⊕⊕◯◯	Critical

Explanations

a. Downgraded one level because most contributing studies raised some concerns regarding insufficient reporting of the randomization process, potential period or carryover effects, and/or the absence of prespecified analysis plans.

b. Downgraded one level because the total information size was limited and the confidence interval ranged from a small to a potentially important benefit. The 95% CI crossed the null after exclusion of Moesgaard et al.

c. Downgraded one level because the information size was limited and the confidence interval included effects ranging from a trivial to a potentially meaningful improvement. The interval crossed the line of no effect under the r = 0.30 assumption and after omission of individual studies.

d. Downgraded one level because substantial unexplained heterogeneity was observed and persisted across alternative correlation assumptions (I^2 approximately 71.9% to 86.4%).

e. Downgraded one level because the available information size was substantially below that required for a precise estimate.

f. Downgraded one level for inconsistency because the direction and timing of the reported RPE effects varied across exercise protocols and assessment points.

g. Downgraded one level for imprecision because the evidence was derived from small studies, several of which did not report complete numerical estimates or formal between-condition comparisons.

h. Downgraded one level for inconsistency because studies reported divergent GI findings and used substantially different symptom definitions, measurement instruments, and assessment time points.

i. Downgraded one level for imprecision because the evidence was derived from small studies and several reports lacked complete condition-specific estimates or formal comparative analyses.

j. Publication bias was not downgraded because no specific evidence of dissemination bias was identified. However, formal assessment was not possible because each quantitative analysis included fewer than 10 studies and the narrative outcomes were not pooled.

k. Downgraded one level because the confidence interval included the possibility of both an appreciable benefit and an appreciable disadvantage of CAF+SB compared with caffeine alone.

l. Downgraded one level because the confidence interval included the possibility of no effect as well as potentially important benefit or disadvantage compared with sodium bicarbonate alone.

Abbreviations: CAF, caffeine; CI, confidence interval; GI, gastrointestinal; GRADE, Grading of Recommendations Assessment, Development and Evaluation; MD, mean difference; PLA, placebo; RoM, ratio of means; RPE, rating of perceived exertion; SB, sodium bicarbonate.

Notes: All included studies used crossover designs. Participant counts represent unique participants and were not doubled across intervention periods. For the GI comparison, two additional studies assessed GI symptoms but did not provide findings attributable specifically to CAF+SB versus placebo and were therefore not included in this comparison-specific GRADE body.
